# Dynamic effects of radioactive iodine therapy on gut microbiota and metabolites in patients with papillary thyroid cancer

**DOI:** 10.1128/spectrum.03237-25

**Published:** 2026-07-10

**Authors:** Yuhui Cao, Xi Zhang, Jie Tan, Yuqi Wang, Qian Guo, Yuan Gao, Jinzhi Chen, Yulong Chen, Keting Tong, Zijian Fu, Linlin Ma, Caixia Wu, Fei Li, Xiaoxi Pang, Meng Liu, Huaiqiu Zhu

**Affiliations:** 1 Department of Nuclear Medicine, Peking University First Hospital 26447, Beijing, China; 2 Department of Biomedical Engineering, College of Future Technology, and Center for Quantitative Biology, Peking University 12465, Beijing, China; 3 Department of Nuclear Medicine, The Second Affiliated Hospital of Anhui Medical University, Anhui, China; Huazhong University of Science and Technology, Wuhan, China

**Keywords:** gut microbiota, metabolism, radioactive iodine therapy, papillary thyroid carcinoma, radiation perturbation, treatment response

## Abstract

The dynamic characteristics of gut microbiome and metabolome during radiopharmaceutical therapy and their correlation with treatment response have not been well clarified. Herein, we conducted a prospective longitudinal study on papillary thyroid carcinoma patients receiving initial radioactive iodine (RAI) treatment. Three fecal samples from each patient were sequentially collected at the day before (Day0), 3 days upon (Day3), and 30 days upon (Day30) receiving RAI (totally 75 samples) for 16S rRNA amplicon sequencing and metabolome profiling. The results revealed transient fluctuations of gut microbiota and metabolites during RAI treatment, as shown by probiotic *Blautia* and anti-inflammatory and antioxidant metabolites first decreased on Day3 and then increased on Day30, while the opposite trends of opportunistic pathogens *Streptococcus* were observed. Meanwhile, correlation analysis showed a complex interplay between the temporal alterations of *Blautia* and the anti-inflammatory and antioxidant metabolites. Furthermore, the variability of intestinal flora and metabolites at multiple time points corresponded to the response to RAI treatment. In conclusion, our findings indicated that internal irradiation could lead to transient fluctuations of gut microbiota and metabolites, suggesting that probiotics might be beneficial for patients receiving RAI. Additionally, we provided a new perspective to screen for noninvasive biomarkers corresponding to the response to RAI treatment.

**IMPORTANCE:**

This study provides the first longitudinal profiling demonstrating that radioactive iodine (RAI) therapy induces transient yet dynamic fluctuations in both the gut microbiome and metabolome. Key beneficial bacteria and anti-inflammatory metabolites were significantly reduced immediately after treatment, followed by a recovery trend. Moreover, patients with different treatment responses showed diverse microbial and metabolic profiles. These findings highlight the potential for probiotic based interventions to maintain gut ecological homeostasis and suggest novel non invasive microbial biomarkers for predicting RAI efficacy.

This study is registered with ClinicalTrials.gov as ChiCTR2100053810.

## INTRODUCTION

The balance of the gut ecosystem may be disrupted by perturbations such as antibiotic therapy, immune checkpoint inhibitors treatment, and radiation, causing significant reductions in functional richness and microbial diversity as well as impacting gut metabolites (1, 2). A systematic review has demonstrated that external ionizing radiation could alter the composition of gut microbiota, which usually embodies the decreased abundance and diversity in gut microbiota, with an increase in harmful bacteria and a decrease in beneficial bacteria (3, 4). Meanwhile, some evidences have indicated that metabolites such as short-chain fatty acids (SCFAs) and taurine can effectively alleviate radiation-induced intestinal injury (5, 6).

Radiopharmaceutical therapy, also known as internal radioactive therapy, exerts its therapeutic effects by directly administrating radionuclides conjugated to the targeted lesion. It differs significantly from external radiotherapy in terms of its therapeutic principle and scope of action (7). With the growing application of radiopharmaceuticals on patients, including iodine-131 (^131^I), lutetium-177 (^177^Lu), and radium-223 (^223^Ra), researchers are increasingly concerned about the influence of internal radiation on the gut ecosystem and metabolites, which have not been well explored currently.

Radioactive iodine (RAI) treatment, one of the most popular internal-radiation therapies, has been widely used in patients with papillary thyroid cancer after radical thyroidectomy (PTCRT). After oral administration, RAI is mainly absorbed and metabolized through the gastrointestinal tract, which is prone to affect the gut microbiome and metabolome. Several recent studies have shown that the gut microbiota or metabolites in patients receiving RAI treatment undergo changes before and after treatment (8–10). Another study found no significant change in the microbiota before and 8–10 days after RAI therapy yet (11). Nevertheless, these studies only observed changes in gut microbiota or/and metabolites at two time points, i.e., before and after treatment, and, therefore, cannot fully reflect the dynamic characteristics in gut microbiota and metabolites during RAI therapy.

On the other hand, recent researches have suggested that gut microbiota and metabolites are associated with radiotherapy responses (12, 13). A multi-omics study revealed that *Bacteroides vulgatus*-mediated nucleotide biosynthesis is associated with neoadjuvant chemoradiotherapy resistance in advanced rectal cancer patients (14). It was also reported that *Bifidobacterium* and *Dorea* might serve as potential predictors for RAI therapeutic response in PTC patients (8). But to the best of our knowledge, the relationship between the dynamic alterations of gut microbiota and metabolites during radiopharmaceutical therapy and the treatment response is not well-known.

Hence, in this real-world and longitudinal self-controlled study, we prospectively performed 16S rRNA amplicon sequencing and metabolome profiling on fecal specimens at multiple time points in order to comprehensively depict the profiles of altered gut microbiota and metabolites in PTCRT patients during RAI therapy. In addition, differences in gut microbiota and metabolites at different time points corresponding to the response to RAI treatment were also preliminarily analyzed.

## MATERIALS AND METHODS

### Study cohort

This study was approved by the Ethics Committee of the Second Affiliated Hospital of Anhui Medical University and registered in the Chinese Clinical Trial Registry (No. ChiCTR2100053810). All participants were willing to participate in this study and had signed the informed consent forms.

We first recruited a total of 31 patients who had been confirmed as PTCRT between April 2019 and September 2019. The inclusion criteria were as follows: (i) 18–65 years old and (ii) qualified with the indication and willing to get initial RAI therapy. The exclusion criteria were as follows: (i) pregnancy, lactation, cigarette smoking, alcohol addiction, hypertension, diabetes mellitus, BMI < 18.5 or BMI > 27; (ii) recent (<3 months prior) use of antibiotics, probiotics, prebiotics, symbiotics, hormonal medication, laxatives, proton pump inhibitors, insulin sensitizers, or traditional Chinese medicine; (iii) known history of disease with an autoimmune component, such as autoimmune thyroid disease, multiple sclerosis, rheumatoid arthritis, irritable bowel syndrome (IBS), or inflammatory bowel disease; and (iv) history of other malignancy besides PTC or any gastrointestinal tract surgery (e.g., gastrectomy, bariatric surgery, colectomy, ileectomy, cholecystectomy, or appendectomy). All patients in this study were from Anhui Province, with similar climates and dietary habits in their residential areas.

### RAI treatment and sample collection

Before RAI treatment initiation, all patients stopped taking levothyroxine or any drugs containing iodine for 4 weeks and followed an iodine-free diet, with serum thyroid stimulating hormone (TSH) concentrations over 30 mIU/L. Patients with no metastasis were treated with RAI of 3,700 MBq (100 mCi) and 5,550 MBq (150 mCi) for someone with metastases.

For each patient, three fecal samples were sequentially collected at the day before RAI administration (Day0), 3 days upon (Day3), and 30 days (Day30) after administration. All fecal samples were aliquoted and stored at −80°C for further 16S rRNA gene sequencing and metabolic profiling analysis. Sequential peripheral blood was collected from patients at different time points, i.e., pre-RAI treatment, 30 days, and 6 months after RAI administration, respectively, in order to assay the levels of thyroglobulin (Tg), thyroglobulin antibody (TgAb), and TSH. RAI-whole body scan was performed at 6 months after RAI treatment.

### Fecal 16S rRNA gene sequencing and bioinformatics analysis

In brief, the hexadecyltrimethylammonium bromide (CTAB) method was used for fecal DNA extraction. The 16S rRNA gene was then amplified by Phusion High-Fidelity PCR Master Mix (New England Biolabs, New England) using specific primers with barcodes. Afterward, sequencing libraries were generated using the TruSeq DNA PCR-Free Sample Preparation Kit (Illumina, USA) and sequenced using the Illumina NovaSeq 6000 platform (Novogene Company, China). Methodological details were provided in Supplemental Materials.

As for bioinformatics analysis, the entire workflow was conducted within the QIIME framework. Raw paired-end reads generated on an Illumina NovaSeq platform were first quality controlled, and singletons were discarded. The reads were then assembled and further quality filtered using fastp (-A -g -q 19 -u 15) and FLASH (V1.2.7) to obtain Clean Tags. Chimeric sequences were identified and removed with VSEARCH (V2.9.0) against the SILVA database, yielding Effective Tags. All Effective Tags were clustered into Operational Taxonomic Units (OTUs) at 97% sequence identity using Uparse (V7.0.1001). After clustering, OTUs that were detected only once across all samples were discarded. For taxonomic annotation, the retained OTU representative sequences were performed with Mothur against the SSU rRNA reference sequences in the SILVA132 database using a confidence threshold of 0.8. OTUs unclassifiable at a given taxonomic level but assignable to a higher level were labeled as “unidentified” and kept at that higher level, while OTUs with no taxonomic assignment were excluded. The raw OTU counts were then rarefied to an even sequencing depth to account for differences in sequencing depth across samples. Alpha (α) diversity indices were calculated based on the rarefied OTU table using R phyloseq (V1.34.0) (15) and microbiome (V1.12.0) packages. Beta (β) diversity was assessed using Bray-Curtis distances calculated from relative abundance data obtained by total sum scaling (TSS) of the raw OTU counts. OTUs shared between different groups were visualized by R Venn Diagram (V1.7.1) and ggplots (V3.3.5) package. Principal Component Analysis (PCA) was performed using R stats package based on the abundance matrices at the genus level. In addition, analysis of similarities (Anosim) function from the R vegan (V2.5-7) package was used to statistically test significant difference in genera composition among different groups.

At the genus level, the Wilcoxon-Mann-Whitney test was used to compare the relative abundances of OTUs between each pair of the three groups (Day0, Day3, and Day30). Genera with *P* < 0.05 in at least one comparison were considered significantly different. For genera significant in multiple comparisons, the smallest *P*-value was used for ranking. The top20 genera with the smallest *P*-values were presented in the heatmap. The Linear Discriminant Analysis (LDA) Effect Size program (LEfSe; http://galaxy.biobakery.org/) (16) was used to identify microbial taxa significantly different in abundance within different groups. Kruskal-Wallis test was used and differential taxa were set as those with *P* < 0.05 and LDA score >3. Meanwhile, the abundance of phyla and genera between any two groups was tested by Wilcoxon rank-sum test, and the *P*-value was adjusted by Bonferroni correction (*Q*). Phyla and genera with *Q* < 0.05 were considered differential. The bacterial correlations were calculated according to the relative abundance of each genus using Python-based SparCC (17) tool based on the SparCC correlation method. The significantly correlated genera (absolute SparCC correlation coefficient >0.60, *Q* < 0.05) were visualized by Cytoscape (V3.8.2) (18). Linear mixed-effects models (LMMs) were employed to account for potential confounding factors. The fixed effects included age, sex, time point, and sequencing batch. Pairwise comparisons were adjusted using Tukey’s method for multiple testing. Principal coordinate analysis (PCoA) based on Bray-Curtis distances was performed to visualize potential batch effects. After that, co-occurrence networks were constructed at the genus level.

### Fecal non-targeted metabolic profiling and bioinformatics analysis

Briefly, frozen fecal samples were thawed and then mixed with 500 μL of an 80% methanol aqueous solution containing 0.1% formic acid and centrifuged at 4°C for 10 min. A quantity of the supernatant was then taken and diluted with mass spectrometry grade water to a methanol content of 53%. After centrifugation at 4°C for 10 min, the supernatant was collected for liquid chromatography-tandem mass spectrometer (LC-MS) analysis (Novogene Company, China).

The raw data files generated by ultra-high performance liquid chromatography-tandem mass spectrometry (UHPLC-MS/MS) were processed using the Compound Discoverer 3.1 (CD3.1, Thermo Fisher) to obtain peak information for each metabolite. And then peaks were matched with the mzCloud (https://www.mzcloud.org/), mzVault, and MassList databases to get the accurate qualitative and relative quantitative results. These metabolites were annotated using the Kyoto Encyclopedia of Genes and Genomes (KEGG) database (https://www. genome.jp/kegg/pathway.html), HMDB database (https://hmdb.ca/metabolites), and LIPID Maps database (http://www.lipidmaps.org/).

After getting the peak intensity matrices, PCA analysis was performed to visualize whether there are differences in metabolite composition between different groups. Then, MetaboAnalyst 5.0 (19) was used to calculate three parameters for the screening of differential metabolites, including variable importance in the projection (VIP), fold change (FC), and *P*-value. The metabolites with VIP > 1, *P*-value < 0.05, and FC > 1.5 or FC < 0.667 were considered to be differential metabolites. Then, these differential metabolites were visualized by Volcano plots using R ggplot2 package, and the top30 differential metabolites were visualized by heatmap using R pheatmap (V1.34.0) package. All samples were processed in a single batch; thus, no batch effect was present. LMMs with age, sex, and time point as fixed effects were applied to longitudinally analyze the changes in key metabolites over time. Pairwise comparisons were performed with Tukey’s correction. Additionally, we performed KEGG enrichment for differential metabolites using MetaboAnalyst 5.0 and visualized the results including the level2 and level3 pathways using the R ggplot2 package.

### Correlation analysis between gut microbiota and metabolites

To assess relationships between gut microbial and metabolic changes across the Day0–Day3 and Day3–Day30 intervals, Spearman correlations were generated using the R psych package (V2.2.3). Change values (Δ1 = Day3 minus Day0, Δ2 = Day30 minus Day3) in bacterial genus abundance and metabolite concentration were calculated for each interval. Additionally, time-lagged correlation analysis was used to assess significantly changing microbiota and metabolite profiles, through cross-correlation of data sets across different time points. If there were more than 20 differential genera or 30 metabolites between two groups, only the top20 genera and top30 metabolites would be selected for heatmap representation using R pheatmap (V1.34.0) package.

### Differences in gut microbiota and metabolites corresponding to the response to RAI therapy

According to the 2015 American Thyroid Association Management Guidelines for Adult Patients with Thyroid Nodules and Differentiated Thyroid Cancer (20), patients with a negative imaging, negative TgAb, and either suppressed Tg < 0.2 ng/mL or stimulated Tg (sTg) < 1 ng/mL at 6 months after RAI treatment are evaluated as excellent response (ER). Those who do not achieve these standards are defined as non-excellent response (NER). Differences in gut microbiota and metabolites between two groups at different time points, namely, Day0, Day3, and Day30, were analyzed and compared, respectively, using R pheatmap (V1.34.0) package.

## RESULTS

### Clinical characteristics of PTCRT patients

**Fig 1:**
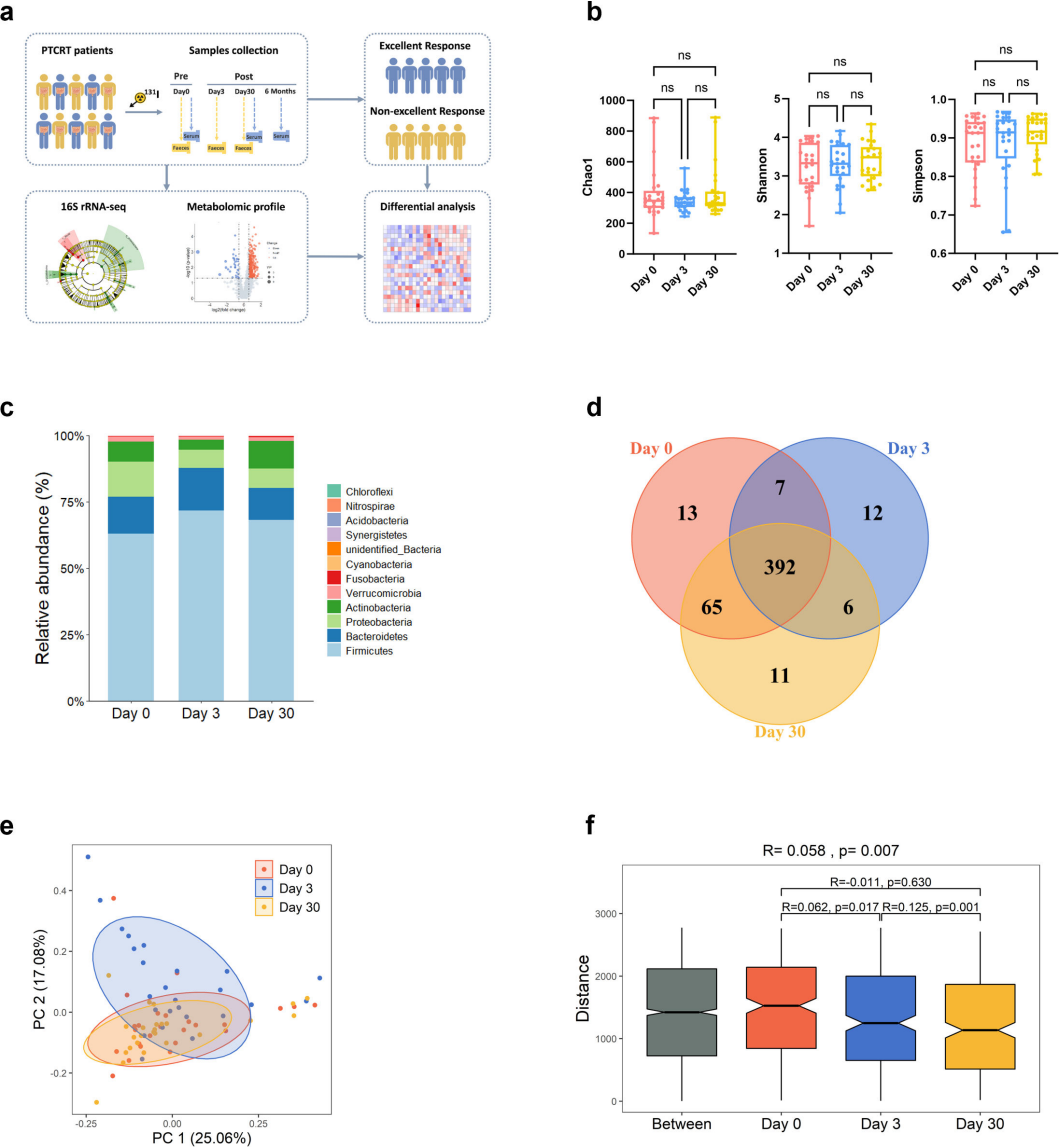
The diversity analysis among three groups. (**a**) Experimental design of the present study. (**b**) The α diversity analysis based on the rarefied absolute abundances; ns indicates not significant (*P* > 0.05). (**c**) Histogram of microbial composition at phylum level. (**d**) Venn diagram based on Operational Taxonomic Units (OTU) tables. (**e**) Principal component analysis (PCA) based on relative abundance matrices at genus level. (**f**) Anosim analysis based on relative abundance matrices at genus level.

Eventually, 25 PTCRT patients who underwent RAI therapy were included in the present study since 6 patients failed to provide complete 3 fecal samples. So, a total of 75 fecal samples of PTCRT patients were collected on the day before the RAI treatment (Day0) and the corresponding time points after the treatment, including 3 days after treatment initiation (Day3) and 30 days upon completion of the treatment (Day30) (Fig. 1a). Baseline clinical characteristics of included patients were summarized in Table 1.

**TABLE 1:** Baseline clinical characteristics of enrolled PTCRT patients^*a*^

Baseline characteristics	PTCRT patients (*n* = 25)
Age, years, mean ± SD	41.60 ± 9.10
Gender, *N* (%)	
Male	7 (28.0%)
Female	18 (72.0%)
Maximum tumor diameter, cm, median (P25, P75)	1.40 (0.80, 1.85)
Local invasion, *N* (%)	
Present	7 (28.0%)
Absent	18 (72.0%)
Lymph node metastasis, *N* (%)	
Present	23 (92.0%)
Absent	2 (8.0%)
Distant metastasis, *N* (%)	
Present	1 (4.0%)
Absent	24 (96.0%)
sTg or TgAb level	
sTg < 1 ng/mL, *N* (%)	4 (16.0%)
sTg 1–10 ng/mL, *N* (%)	5 (20.0%)
sTg > 10 ng/mL or rising TgAb level, *N* (%)	16 (64.0%)

^*a*^ PTCRT, papillary thyroid carcinoma after radical thyroidectomy; sTg, stimulated thyroglobulin; TgAb, thyroglobulin antibody.

### Alteration of gut microbiota in PTCRT patients during RAI therapy

To analyze the alteration in gut microbiota during RAI treatment, we evaluated the microbial diversity and composition across different time points Day0, Day3, and Day30.

Regardless of the genus or phylum level, there were no significant differences in the α-diversity indices of gut microbiota among three groups (Fig. 1b), indicating that RAI treatment did not lead to the collapse of the overall microbial community structure. In terms of microbial composition at phylum level, *Firmicutes*, *Bacteroidetes*, *Proteobacteria*, together with *Actinobacteria* were the four predominant phyla in three groups (Fig. 1c). The Venn diagram (Fig. 1d) and the unconstrained PCA (Fig. 1e) based on OTU tables indicated better similarity between the microbiome on Day0 and Day30, while there was dissimilarity between Day3 and Day0 or Day30, respectively. The result of Anosim analysis demonstrated that there was a significant difference in β diversity between Day0 and Day3 (*P* = 0.017, *R* = 0.062) and Day3 and Day30 (*P* = 0.001, *R* = 0.125), respectively, whereas there was no significant difference between Day0 and Day30 (*P* = 0.63, *R* = −0.011) (Fig. 1f).

**Fig 2:**
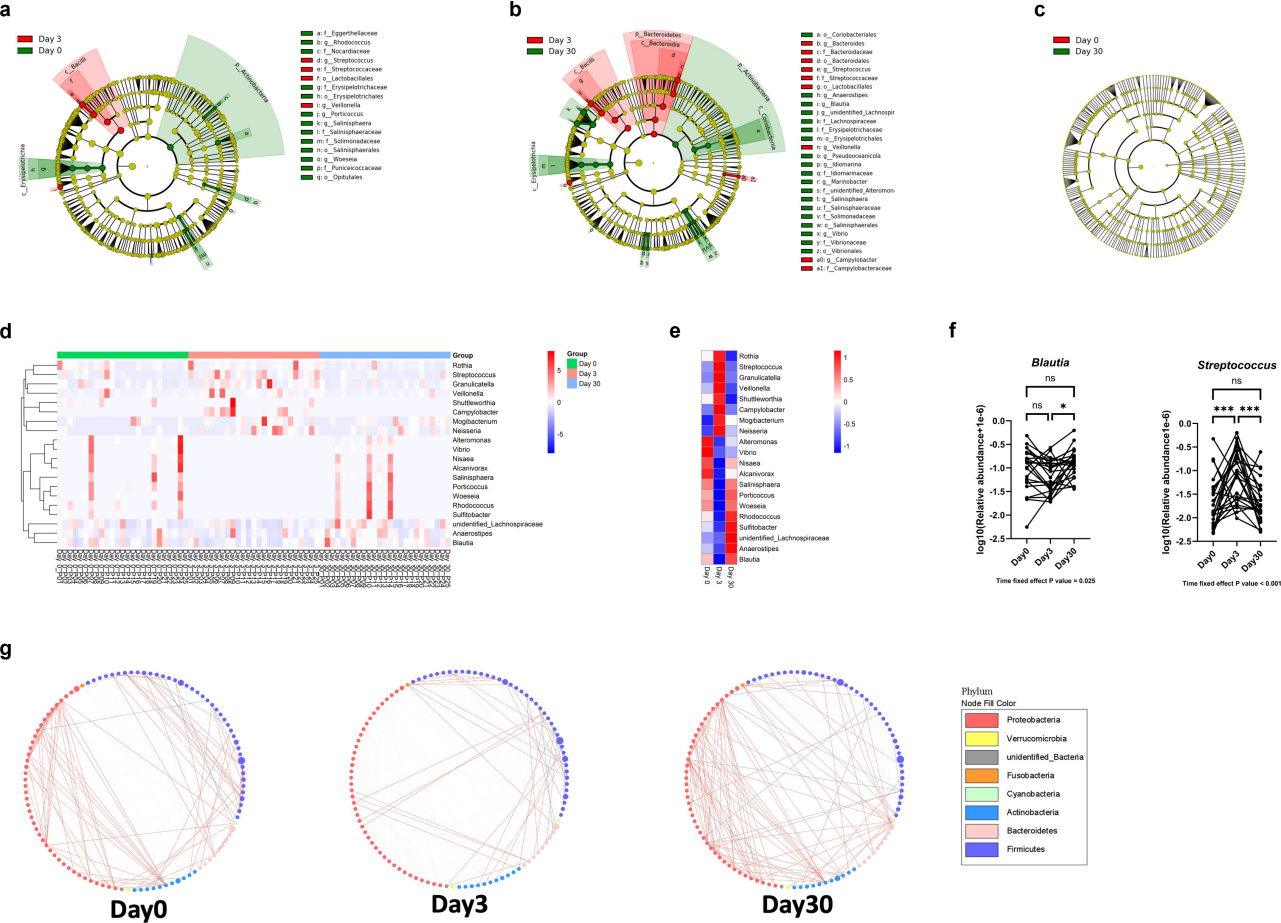
Fluctuating changes of gut microbiota in papillary thyroid cancer after radical thyroidectomy (PTCRT) patients during RAI treatment. (**a**) Linear discriminant analysis (LDA) effect size analysis (LEfSe) in cladogram between Day0 and Day3. (**b**) LEfSe in cladogram between Day3 and Day30. (**c**) LEfSe in cladogram between Day0 and Day30. (**d**) Heatmap of the relative abundances of top20 different flora at genus level among samples. (**e**) Heatmap of the relative abundances of top20 different flora at genus level among groups. (**f**) Violin plots of the relative abundances of *Blautia* and *Streptococcus* across different time points. *P*-values were calculated using linear mixed-effects models (LMMs) with adjustment for age, sex, time point, and sequencing batch. (**g**) Co-occurrence networks based on the Spearman correlation algorithms among three groups. Node sizes represent the relative abundance of each genus. Edges between nodes represent significant correlations, with red and blue lines denoting positive and negative correlations, respectively. ns indicates not significant (*P* > 0.05), *P < 0.05, and ***P < 0.001.

To further observe the specific differences in gut microbiota during RAI treatment, we performed LEfSe analysis. The results showed a significant decrease in *Actinobacteria*, *Erysipelotrichaceae*, *Solimonadaceae,* and *Salinisphaera*, and a drastic increase in the harmful bacteria of *Streptococcus* and *Veillonella* on Day3 compared to Day0 (Fig. 2a), while these altered bacteria (increased or decreased) returned to their original abundance on Day30 (Fig. 2b). Between Day0 and Day30, there were no significant differences in gut microbiota (Fig. 2c). The heatmap of the top 20 differential microbes in genus level also showed the fluctuations of some genus among samples (Fig. 2d) and groups (Fig. 2e), including *Woeseia*, *Salinisphaere*, *Porticoccus*, *Blautia*, *Anaerostipes*, *Rhodococcus*, and *Alcanivorax* decreased on Day3, but enriched on Day30. Opposite trends were observed in *Rothia*, *Shuttleworthia*, *Veillonella*, *Streptococcus*, *Granulicatella*, *Campylobacter*, and *Neisseria*. Although a moderate batch effect was observed for some taxa (Fig. S1, Table S1), Fig. 2f demonstrated a significant fixed effect of time point on changing patterns of *Blautia* and *Streptococcus* (*P* = 0.025 and *P* < 0.001, respectively) after adjusting for potential confounding factors. For *Streptococcus* in particular, a significant trend of increasing and then decreasing was observed across both phases, with *P* < 0.001 for each pairwise comparison.

In order to explore the symbiotic and competitive interactions within the microbial community during RAI treatment, we mapped the co-occurrence networks (Fig. 2g). Quantitative network properties were presented in Table S2. Compared to the baseline, the microbial network on Day3 became markedly sparser, evidenced by a visible decrease in both average degree and graph density, which signaled a sharp reduction of genera interactions. The structural fragmentation was accompanied by extended network diameter and average path length, indicating that information transfer within the community had become less efficient. A concurrent substantial drop in global transitivity further pointed to weakened stability. However, all these network properties robustly rebounded on Day30, with several reaching levels that surpassed the baseline. This indicates that microbial interactions had reconstituted into a stabilized network structure.

The above observations suggested that the gut microbiota exhibited a characteristic of “fluctuating changes” during RAI treatment. In other words, there was a transient and significant change in the richness, composition, and interaction of gut microbiota on Day3 after RAI treatment compared to pre-treatment (Day0), but followed by a reversion to the pre-treatment state by Day30.

### Alteration of gut metabolome in PTCRT patients during RAI therapy

**Fig 3:**
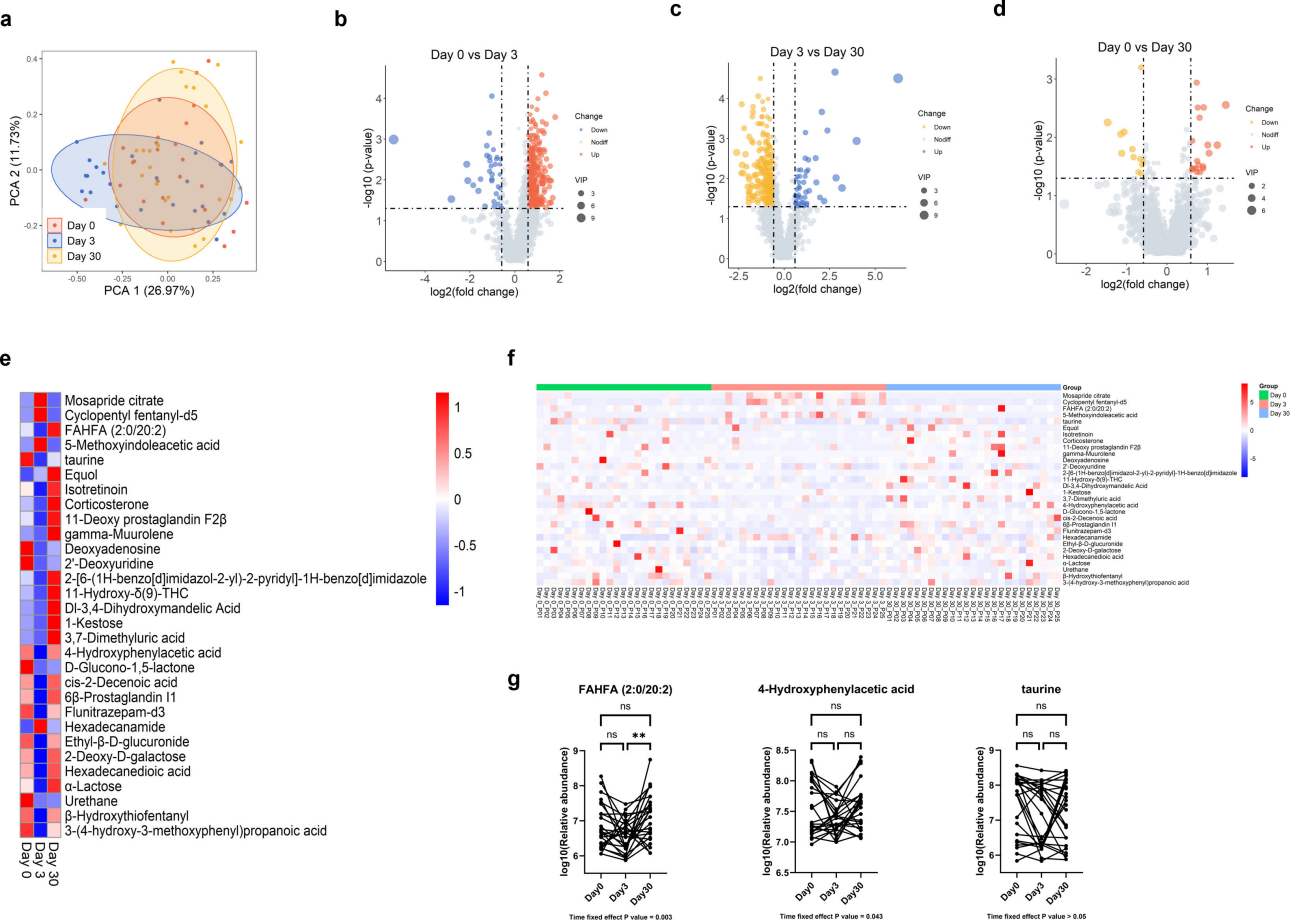
Alterations of metabolites in papillary thyroid cancer after radical thyroidectomy (PTCRT) patients during RAI therapy. (**a**) Principal component analysis (PCA) of gut metabolites. (**b–d**) Volcano plots of differential metabolites among three groups. (**e**) Heatmap of the top30 differential metabolites among groups. (**f**) Heatmap of the top30 differential metabolites among samples. (**g**) Violin plots of the relative abundances of Fatty acid esters of hydroxy fatty acids (2:0/20:2), 4-hydroxyphenylacetic acid and taurine across different time points. *P*-values were calculated using linear mixed-effects models (LMMs) with adjustment for age, sex, and time point. ns indicates not significant (*P* > 0.05) and **P < 0.01.

The alterations of gut metabolome across different time points during RAI treatment were also analyzed. The PCA analysis depicted that in the negative polarity mode, there were significant differences in gut metabolites between Day3 and Day0, and between Day3 and Day30, respectively, while there was no significant difference between Day0 and Day30 (Fig. 3a). Differential metabolites were shown in volcano plots, indicating that there were numerous differential metabolites between Day0 and Day3, and between Day3 and Day30, but only a small number of differential metabolites existed between Day0 and Day30 (Fig. 3b and d).

To understand the specifically altered metabolites during RAI therapy, partial least squares-discriminant analysis (PLS-DA) of gut microbial profiles from three groups was performed. The top30 differential metabolites among groups (Fig. 3e) and samples (Fig. 3f) were listed in the heatmap. Most of the metabolites showed a decline on Day3 and return to or even exceed baseline levels on Day30. These metabolites included 2-Deoxy-D-galactose, cis-2-Decenoic acid, Hexadecanedioic acid, 6β-Prostaglandin l1 (6β-PGl1), Fatty acid esters of hydroxy fatty acids (FAHFA) (2:0/20:2), 4-Hydroxyphenylacetic acid (4-HPA), β-Hydroxythiofentanyl, α-Lactose, Isotretinoin, Flunitrazepam-d3, Deoxyadenosine, 2′-Deoxyuridine and taurine. A few metabolites enriched on Day3, and decreased on Day30, such as Hexadecanamide, Cyclopentyl fentanyl-d5, 5-Methoxyindoleacetic acid, and Mosapride citrate. Distinguishably, equol consistently showed an upward trend during the course of RAI therapy. The biphasic trends in certain anti-inflammatory metabolites (FAHFA (2:0/20:2), 4-HPA and taurine) were presented in Fig. 3g; Table S3. The changes in both FAHFA (2:0/20:2) and 4-HPA were characterized by a significant time fixed effect (*P* = 0.003 and 0.043, respectively), with a notable decrease from Day0 to Day3 followed by a further rally from Day3 to Day30. Taurine displayed an analogous trajectory, yet its time main effect did not reach statistical significance in the LMM model.

**Fig 4:**
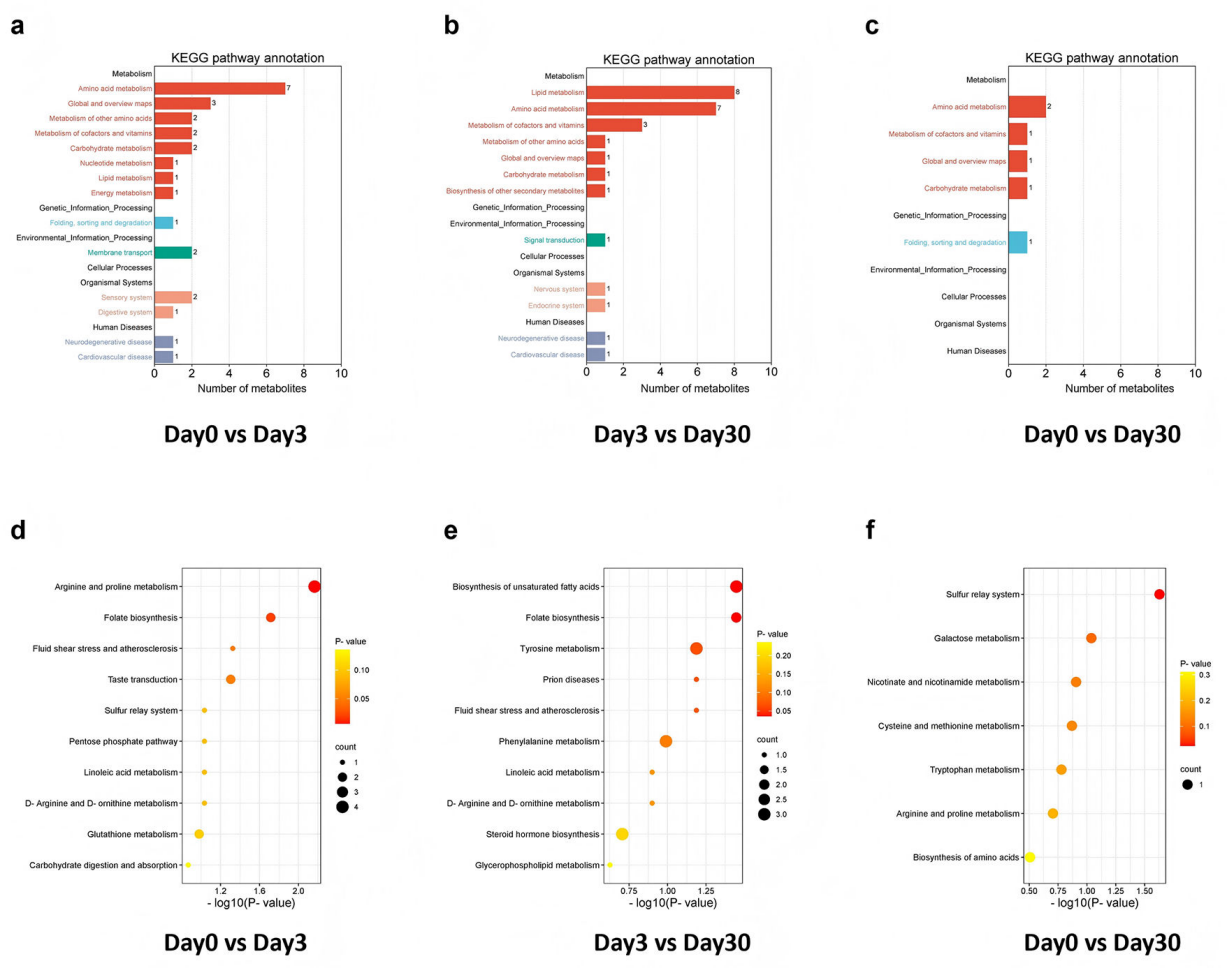
The functions of differential metabolites and metabolic pathways. (**a–c**) The catalogs of KEGG pathways ranked at the number of differential metabolites. (**d–f**) The top10 enriched metabolic pathways of differential metabolites.

The differential metabolites were mapped to the KEGG database for the enrichment of functional modules (Fig. 4a through c) and metabolic pathways (Fig. 4d through f). The main enrichment catalog of differential metabolites between Day0 and Day3 was amino acid metabolism, overlapped with that between Day3 and Day30. Besides, there were most differential metabolites annotated as lipid metabolism between Day3 and Day30 but few annotated between Day0 and Day30. The enriched metabolic pathways of significance between Day0 and Day3 were arginine and proline metabolism, folate biosynthesis, fluid shear stress and atherosclerosis, and taste transduction, whereas those between Day3 and Day30 were Biosynthesis of unsaturated fatty acids and Folate biosynthesis. In addition, there was only one significant metabolic pathway between Day0 and Day30, namely, Sulfur relay system. Specific differential metabolites in each enriched metabolic pathway of significance were provided in Fig. S2 to S4.

### Relationships between the alterations in gut microbiome and metabolome

**Fig 5:**
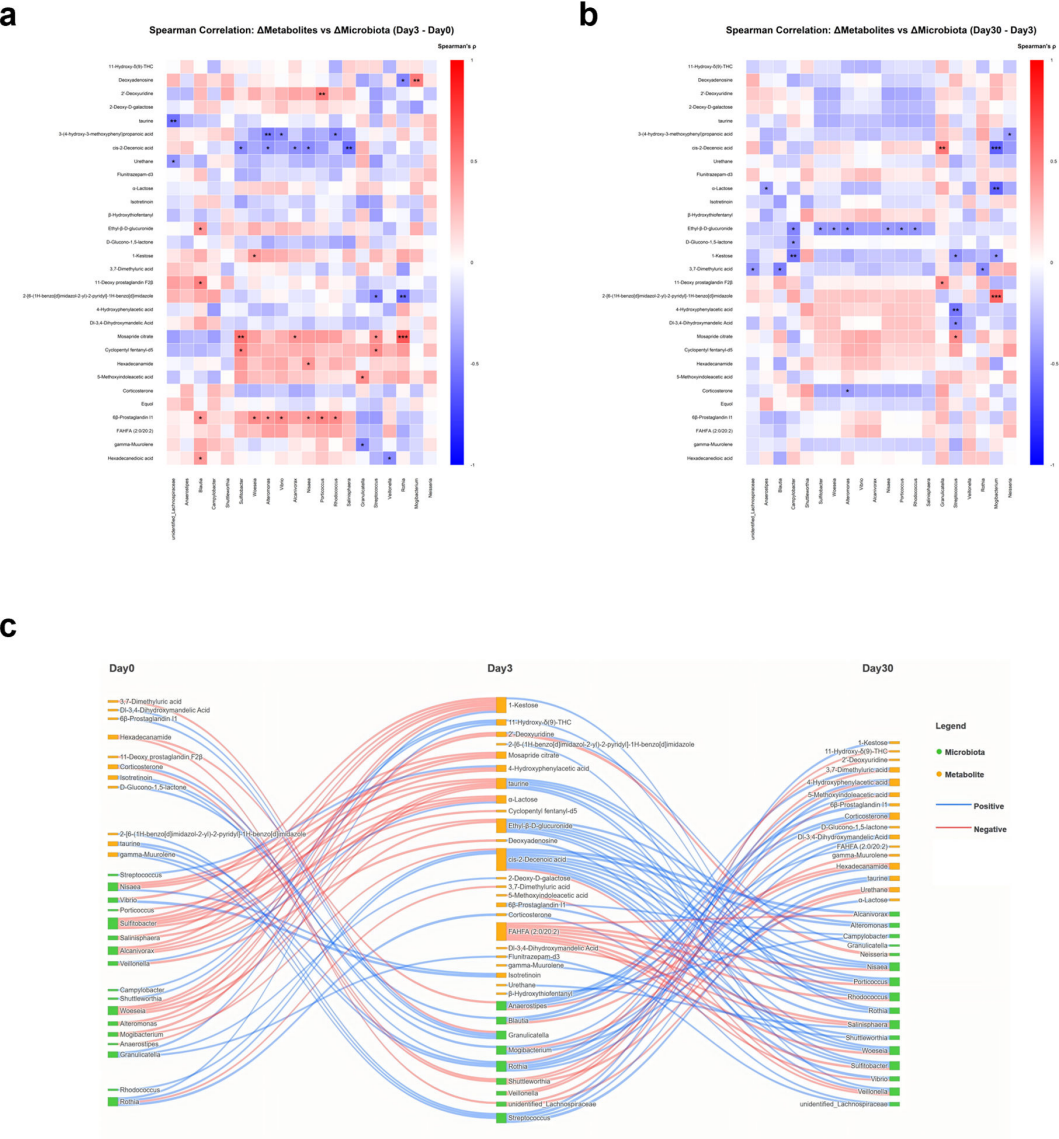
Relationships between genera and metabolites using Spearman correlation analysis. Heatmap showing the correlations between alterations in microbial genera and metabolites from Day0 to Day3 (**a**), and from Day3 to Day30 (**b**). Sankey diagram of time-lagged correlation between microbial genera across three time points (**c**). The correlation effect is indicated by a color gradient from blue (negative correlation) to red (positive correlation). *P < 0.05, **P < 0.01, ***P < 0.001.

To explore the potential relationships between the alterations in gut microbiome and metabolome, a correlation matrix was generated using Spearman correlation between Day0 and Day3 (Fig. 5a) and Day3 and Day30 (Fig. 5b), respectively. The outcome determined 36 significant Δmicrobiota–Δmetabolite correlations between Day0 and Day3, 25 significant correlations between Day3 and Day30. In particular, the change of *Blautia* showed a significant positive association with the decrease of Ethyl-β-D-glucuronide, 11-Deoxy prostaglandin F2β (PGF2β), 6β-PGI1 and Hexadecanedioic acid from Day0 to Day3, and a negative association with 3,7-Dimethyluric acid from Day3 to Day30. The variation of 4-HPA correlated negatively with *Streptococcus* from Day3 to Day30. Both between Day3 and Day0 or Day30, strong positive correlations were observed between *Streptococcus* and mosapride citrate.

The time-lagged correlation analysis (Fig. 5c) revealed a substantial number of significant microbiota-metabolite pairs across different time points, with a notable directional distribution. Namely, 40 significant correlations were identified between Day0 microbiota and Day3 metabolite, whereas only 16 were found between Day0 metabolite and Day3 microbiota. Conversely, 27 significant pairs were observed between Day3 microbiota and Day30 metabolite, compared to 45 between Day3 metabolite and Day30 microbiota. These included a positive correlation between *Streptococcus* at Day0 and 4-hydroxyphenylacetic acid at Day3, as well as between *Streptococcus* at Day3 and 4-hydroxyphenylacetic acid at Day30. Furthermore, *Blautia* at Day3 was positively correlated with D-glucono-1,5-lactone at Day0, and with both 3,7-dimethyluric acid and corticosterone at Day30.

### Distinct gut microbiota and metabolome between ER and NER patients

**Fig 6:**
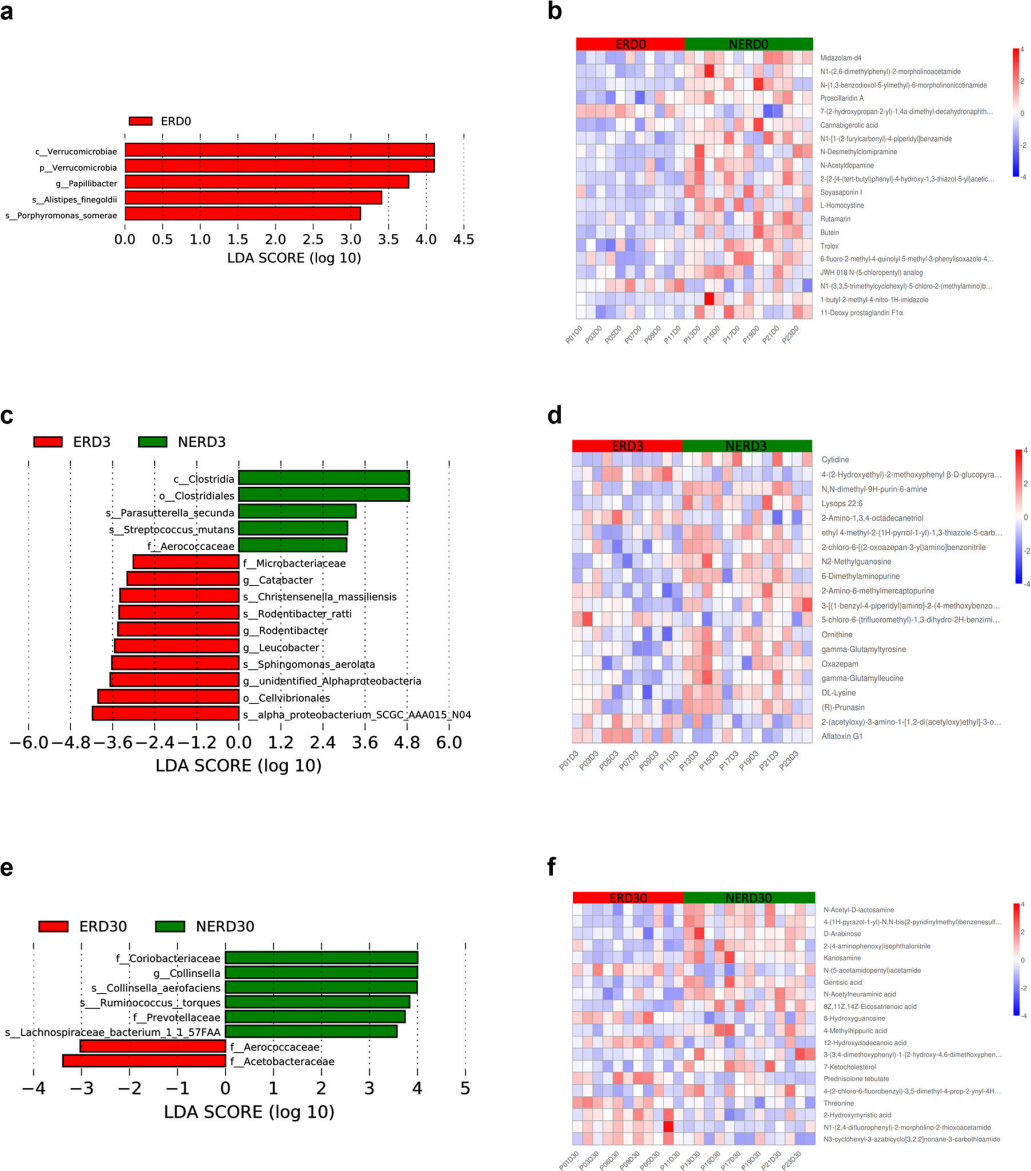
Differential gut microbiota and metabolites between excellent response (ER) and non-excellent response (NER) group. (**a, c, and e**) Linear discriminant analysis (LDA) effect size analysis (LEfSe) of gut microbiota between ER and NER on Day0 (**a**), Day3 (**c**), and Day30 (**e**); all listed taxa were significantly (Kruskal-Wallis test, *P* < 0.05, LDA score > 3) enriched for their respective groups. (**b, d, and f**) Heatmap of the top20 differential metabolites between ER and NER on Day0 (**b**), Day3 (**d**), and Day30 (**f**).

According to the treatment response criteria mentioned above, 11 patients were defined as ER and 13 were NER (1 patient was lost in follow-up). The differential compositions of gut microbiota (Fig. 6a, c and e) and differential metabolites (Fig. 6b, d and f) between ER and NER group at different time points were observed.

On Day0, there were higher richnesses of *Verrucomicrobia, Papillibacter,* and *Alistipes finegoldii* as well as lower levels of N-Desmethylclomipramine, N-Acetyldopamine, and L-Homocystine in ER patients. On Day3, the relative abundances of *Alphaproteobacteria*, *Leucobacter,* and *Rodentibacter ratti* were higher in ER patients, while *Clostridia* which can produce SCFAs enriched in NER patients. And some nucleotide components (cytidine, N2-Methylguanosine and 6-Dimethylaminopurine) and amino acids (Ornithine and DL-Lysine) enriched in NER patients. On Day30, the result showed increments of *Aerococcaceae* and *Acetobacteraceae* in ER group, and some harmful bacteria such as *Collinsella aerofaciens* and *Ruminococcus torques* enriched in NER group. The levels of 8-Hydroxyguanosine, Threonine, and Hydroxy fatty acids (HFAs) including 12-Hydroxydodecanoic acid and 2-Hydroxymyristic acid in ER group were higher than those in NER group, while the levels of some carbohydrates including N-Acetyl-D-lactosamine, D-Arabinose, and N-Acetylneuraminic acid were lower.

## DISCUSSION

At present, the altered characteristics of gut microbiota and metabolites during radiopharmaceutical therapy have not been well elucidated. In particular, sampling at multiple time points throughout the course of treatment is critical for accurately identifying changes in the microbiome and metabolites, but this has been overlooked in previous studies. To address these issues, we conducted a prospective longitudinal study using RAI-treated PTCRT patients as a model to decipher the profiles of altered gut microbiota and metabolites during RAI therapy and explore their potential association with response to RAI treatment.

First, we focused on analyzing the dynamic characteristics of gut microbiome during RAI treatment (Day0, Day3, and Day30). Our results showed that the similarity in microbiome composition and microbiota connectivity between Day0 and Day30 was better compared to that between Day0 and Day3, as well as Day3 and Day30. Meanwhile, the composition of microbes exhibited a characteristic of “fluctuating changes.” Specifically, the harmful bacteria (such as *Streptococcus* and *Campylobacter*) significantly increased while the beneficial bacteria (such as *Blautia*) significantly decreased on Day3, but all returned to baseline levels (Day0) on Day30. Similar fluctuations in the gut microbiota have been found in antibiotic exposure study, demonstrating that although the gut microbiome is constantly exposed to external exposure, it still has the ability to restore equilibrium after external challenges (21). Our findings provided the evidence that there are reversible effects of internal irradiation (e.g., radiopharmaceutical therapy) on the gut microbiome.

Furthermore, our study showed genus *Blautia*, which is belonging to family *Lachnospiraceae* known for the synthesis of SCFAs by fermentation of dietary polysaccharides (22, 23), decreased on Day3. As a potential probiotic (24), *Blautia* can not only generate a variety of antimicrobials but also produce SCFAs and upregulate regulatory T (Treg) cells, thereby controlling intestinal inflammatory response (25). Considering its probiotic potential, *Blautia* supplementation for patients post RAI treatment may be a feasible strategy for mitigating intestinal adverse events. We also observed fluctuations in other SCFAs-producing bacteria. For instance, *Anaerostipes* decreased on Day3 and then rebounded, whereas *Veillonella* and *Streptococcus* occurred an inverse change. *Streptococcus*, one of the major enteral opportunistic pathogens (26), increased significantly on Day3, which is consistent with previous studies investigating changes in intestinal flora after abdominal and pelvic radiotherapy (27, 28). Nevertheless, the LC-MS metabolic profiles depicted no significant difference in the content of SCFAs among the three groups. Also, Spearman correlation analysis showed no correlation between SCFAs and SCFAs-producing bacteria. Such results may be due to the fact that the gut is a complicated micro-ecosystem, which compensates for the functional niche following a disturbance, such as a decline in some SCFAs-producing bacteria and an increase in others.

Meanwhile, the fluctuation of the abundances of numerous metabolites was first observed in our study. Several substances with antioxidant and anti-inflammatory functions (such as FAHFA [29], 4-HPA [30], and taurine [6]) decreased on Day3 but recovered on Day30 (the lack of a significant fixed effect of taurine may be attributed to outliers in limited samples). In addition, compounds related to DNA repairment (deoxyadenosine and 2′-deoxyuridine) exhibited a similar fluctuating pattern. We speculated that the “fluctuating changes” in these substances might be due to their short-term over-depletion by the ionizing radiation effects generated by RAI. It is well known that mitochondrial DNA is a sensitive target for radiation-induced damage that will lead to the generation of high-level reactive oxygen species (ROS) (31). Taurine is produced from sulfur-containing amino acid, which not only reduces the generation of ROS by adjusting biosynthesis and mitochondrial function but also suppresses inflammation via inhibiting cytokines (6). Additionally, both FAHFA and taurine can activate the Nrf2 signaling pathway to induce the expression of downstream cell protective genes, so as to mitigate radiation-induced injury (32, 33). These findings suggested that short-term supplementation of anti-inflammatory and antioxidant metabolites might alleviate radiation damage during internal radiation therapy.

According to KEGG enrichment analysis, tetrahydrobiopterin (BH4) was upregulated in the folate biosynthesis pathway while sepiapterin and 7,8-dihydrobiopterin (7,8-BH2) were downregulated on Day3 compared to both Day0 and Day30. Given that BH4 can be synthesized from sepiapterin and 7,8-BH2 via the salvage pathway (34), its increase might reflect intestinal stress and repair caused by proinflammatory factor (e.g., tumor necrosis factor-α, interferon-γ, and interleukin-1) and ROS (34). The most significantly altered pathway between Day0 and Day3 was arginine and proline metabolism, which contributes to damaged tissue repair and played a protective role in radiation-induced enteritis (35) through mediators such as nitric oxide, polyamines, and proline (36). In addition, the biosynthesis of unsaturated fatty acids also showed significant difference described as depleted omega-3 polyunsaturated fatty acids (ω−3 PUFAs) (eicosapentaenoic acid) and heightened ω−6 PUFAs (linoleic acid) on Day3 compared to Day30. Since ω−3 and ω−6 PUFAs exert anti-inflammatory and pro-inflammatory effects, respectively (37), it might explain the intestinal inflammation due to overall perturbations in host immunity and gut microbes (38) after RAI administration. In general, most of differential metabolites observed in this study were annotated as amino acid and lipid metabolism, revealing the major alterations of metabolic profiles after internal RAI irradiation. On the other hand, previous studies have implicated that radiation exposure could affect cellular energy metabolism and adenosine triphosphate production (39). Nevertheless, these mentioned pathways were not enriched in this study, which might be attributed to less damage to cell mitochondria by RAI compared to external radiotherapy.

Among the considerable relationships between the gut microbiome changes and metabolic products, we noticed that the change of *Blautia* exhibited positive correlations with 11-Deoxy PGF2β, 6β-PGI1. PGs exhibit a dual role in inflammation, encompassing both pro-inflammatory subtypes like PGE₂ and anti-inflammatory, tissue-protective substances like PGI₂ (40). Although 11-Deoxy PGF2β and 6β-PGI1 are not typical physiological PGs, their synchronous decline with the probiotic *Blautia* from Day0 to Day3 suggests that *Blautia* may contribute to the homeostasis of PG-associated lipid metabolism. The reduction of *Blautia* may thereby compromise the modulation of intestinal and systemic inflammation during the early phase receiving RAI. From Day3 to Day30, the inverse correlation between the increase in *Blautia* and 3,7-dimethyluric acid may reveal a collaboration between the gut microbiota and host metabolism when combating RAI-induced oxidative stress. As a 1,3,7-trimethyluric acid analog, 3,7-dimethyluric acid can directly scavenge ROS and inhibit lipid peroxidation (41), and its elevated level may represent a compensatory host response to radiation-induced oxidative damage. *Blautia* contributes to microbial intestinal defense through the production of SCFAs. The negative correlation suggests that when microbial defense is more effective, the demand for the antioxidant metabolites may be relatively reduced. Conversely, a less robust microbial response would necessitate a stronger compensatory antioxidant mobilization by the host. This interplay underscores a coordinated adaptation within the host-microbiota symbiosis to restore homeostasis following radiation perturbation. From this perspective, supplementing anti-inflammatory and antioxidant metabolites might be a logical approach to promote the restoration of normal intestinal environment, but further research is needed.

The time-lagged correlation analysis revealed the Day0microbiota–Day3metabolite associations predominated in the acute phase post-therapy. This indicates that gut microbial composition preceded or potentially shaped the subsequent metabolic profile during the initial perturbation. Interestingly, this directional pattern reversed in the later recovery period, with Day3metabolite–Day30microbiota pairs being more numerous. The shift may suggest that the earlier metabolic alterations thereafter played a decisive role in driving microbial community reconstruction. Intervention strategies targeting the gut microbiota in the acute phase and modulating the metabolic landscape during recovery period may promote the intestinal ecosystem rehabilitation.

Furthermore, we analyzed the difference in composition of gut microbiota and metabolites between ER and NER patients at different time points, respectively. On Day0, there were increased *Alistipes finegoldii* in ER patients, which has been identified as colitis-protective species in mice models (42). On Day3, *Clostridia* was found to enrich in NER patients. A published literature reported a *Clostridia*-rich microbiota as well as more bile acids (BAs) such as glycochenodeoxycholic acid and glycoursodeoxycholic acid in IBS patients (43), which reached an agreement with our observation (although these two BAs were not included in the top20 metabolites). Given that *Clostridia*-derived BAs could promote intestinal inflammation (43), we assumed that precisely identifying and targeting these specific BAs-producing *Clostridial* subgroups could enhance the treatment efficacy of RAI. In addition, more nucleotide components and amino acids were discovered in NER group, which might imply an intrinsic relationship between the extent of intestinal cellular damage and the response to RAI treatment. On Day30, it is important to note the high levels of HFAs in ER group and carbohydrates in NER group, respectively. As intermediates of bacteria-mediated lipid metabolism, HFAs could support the role of fatty acids in modifying the host gut environment (44). Enteric microorganisms, including some harmful bacteria observed in NER group, could hydrolyze carbohydrates as metabolic substrates, which aggravated intestinal dysfunction and further impaired the therapeutic effect (45). Altogether, these observed differential microbiota or metabolites at different time points might be promising noninvasive biomarkers to predict the response to RAI at early stage and adjust subsequent treatment strategy in time, which requires further research in the future.

Certainly, there are some limitations in the current study. First, although this study is the first of its kind to characterize the dynamic changes in gut microbiota and metabolites under internal radiation therapy, further validation of the findings is needed as it is a single-center, small-scale study. Second, this observational study lacks the elucidation of specific molecular mechanism or causal relationship, which requires subsequent intervention experiments. Third, this study only observed the differences in gut microbiota and metabolites between the treatment responsive and non-responsive groups at different time points. In the future, a predictive model for responses to RAI treatment based on gut microbiome and metabolism could be further established systematically in order to provide individualized precision therapy for PTCRT patients. Last but not least, based on our findings, we consider that the alteration of gut ecosystem imposed by RAI therapy may not be as drastic as external radiotherapy from, and assume that this alteration is not a reconstruction of another unhealthy stable state, but rather a transient fluctuation. So, the underlying mechanism of the incompatible influence of external and internal radiation imposed on gut microbiome deserves further investigation.

In conclusion, the dynamic profiles of gut microbiota and metabolites in PTCRT patients during RAI treatment were depicted in our study for the first time. This characterization could be summarized as “fluctuating alterations,” where some SCFAs-producing beneficial bacteria and several antioxidant and anti-inflammatory substances reduced transiently, but opportunistic pathogens increased temporarily, suggesting that decent amounts of probiotics (e.g., *Blautia*) and anti-inflammatory and antioxidant metabolites might be beneficial for patients undergoing RAI treatment. Additionally, we provide a basis for screening noninvasive biomarkers at different times corresponding to the responses to RAI treatment from a new perspective of gut microbiome and metabolome.

## Supplementary Material

Reviewer comments

## Data Availability

Fecal 16S rRNA gene sequencing data in this study are available in the National Center for Biotechnology Information (NCBI), under the BioProject ID PRJNA1107925.

## SUPPLEMENTAL MATERIAL

Supplemental material spectrum.03237-25-s0001.docx Supplemental text, figures, and tables.

OPEN PEER REVIEW reviewer-comments.pdf An accounting of the reviewer comments and feedback.

ASM does not own the copyrights to Supplemental Material that may be linked to, or accessed through, an article. The authors have granted ASM a non-exclusive, world-wide license to publish the Supplemental Material files. Please contact the corresponding author directly for reuse.
