# Supplementary material for: Dynamic effects of radioactive iodine therapy on gut microbiota and metabolites in patients with papillary thyroid cancer
Source: Microbiol Spectr. 2026 Jul 10;14(8):e03237-25. doi: 10.1128/spectrum.03237-25 (PMC13436337; doi:10.1128/spectrum.03237-25)
Supplement: Supplementary file 1 [file reviewer-comments.pdf]

AMERICAN SOCIETY FOR MICROBIOLOGY | Microbiology Spectrum

Dynamic effects of radioactive iodine therapy on gut microbiota and metabolites in patients with papillary thyroid cancer

Yuhui Cao, Xi Zhang, Jie Tan, Yuqi Wang, Qian Guo, Yuan Gao, Jinzhi Chen, Yulong Chen, Keting Tong, Zijian Fu, Linlin Ma, Caixia Wu, Fei Li, Xiaoxi Pang, Meng Liu, and Huaiqiu Zhu

Corresponding Author(s): Meng Liu, Peking University First Hospital

Review Timeline:

Submission Date:	November 1, 2025
Editorial Decision:	December 12, 2025
Revision Received:	February 8, 2026
Editorial Decision:	March 18, 2026
Revision Received:	April 23, 2026
Accepted:	April 23, 2026

Editor: Wei-Hua Chen

Reviewer(s): The reviewers have opted to remain anonymous.

Transaction Report:

(Note: With the exception of the correction of typographical or spelling errors that could be a source of ambiguity, letters and reports are not edited. The original formatting of letters and referee reports may not be reflected in this compilation.)

DOI: https://doi.org/10.1128/spectrum.03237-25

1st Editorial Decision December 12, 2025

Re: Spectrum03237-25 (Dynamic effects of radioactive iodine therapy on gut microbiota and metabolites in patients with papillary thyroid cancer)

Dear Prof. Meng Liu:

Thank you for the privilege of reviewing your work. Below you will find my comments, instructions from the Spectrum editorial office, and the reviewer comments.

Please return the manuscript within 60 days; if you cannot complete the modification within this time period, please contact me. If you do not wish to modify the manuscript and prefer to submit it to another journal, notify me immediately so that the manuscript may be formally withdrawn from consideration by Spectrum.

Revision Guidelines
To submit your modified manuscript, log into the submission site at https://spectrum.msubmit.net/cgi-bin/main.plex. Go to Author Tasks and click the appropriate manuscript title to begin. The information you entered when you first submitted the paper will be displayed; update this as necessary. Note the following requirements:

- Upload point-by-point responses to the issues raised by the reviewers in a file named "Response to Reviewers," NOT in your cover letter.
- Upload a compare copy of the manuscript (without figures) as a "Marked-Up Manuscript" file.
- Upload a clean .DOC/.DOCX version of the revised manuscript and remove the previous version.
- Each figure must be uploaded as a separate, editable, high-resolution file (TIFF or EPS preferred), and any multipanel figures must be assembled into one file.
- Any supplemental material intended for posting by ASM should be uploaded with their legends separate from the main manuscript. You can combine all supplemental material into one file (preferred) or split it into a maximum of 10 files with all associated legends included.

For complete guidelines on revision requirements, see our Submission and Review Process webpage. Submission of a paper that does not conform to guidelines may delay acceptance of your manuscript.

Data availability: ASM policy requires that data be available to the public upon online posting of the article, so please verify all links to sequence records, if present, and make sure that each number retrieves the full record of the data. If a new accession number is not linked or a link is broken, provide Spectrum production staff with the correct URL for the record. If the accession numbers for new data are not publicly accessible before the expected online posting of the article, publication may be delayed; please contact production staff (Spectrum@asmusa.org) immediately with the expected release date.

Publication Fees: For information on publication fees and which article types are subject to charges, visit our website. If your manuscript is accepted for publication and any fees apply, you will be contacted separately about payment during the production process; please follow the instructions in that e-mail. Arrangements for payment must be made before your article is published.

ASM Membership: Corresponding authors may join or renew ASM membership to obtain discounts on publication fees. Need to upgrade your membership level? Please contact Customer Service at Service@asmusa.org.

The ASM Journals program strives for constant improvement in our submission and publication process. Please tell us how we can improve your experience by taking this quick Author Survey.

Thank you for submitting your paper to Spectrum.

Sincerely,
Wei-Hua Chen
Editor
Microbiology Spectrum

Reviewer #1 (Comments for the Author):

The manuscript is not adequately prepared for scientific evaluation by this journal. In particular, the Results and Discussion sections require substantial revision before the manuscript can be considered for review. Additionally, the Results section must be rewritten, as technical findings are described without biological context. The manuscript also contains multiple errors and inconsistencies in the authors' claims. Furthermore, the Methods section omits key technical steps in sequencing and data analysis. Overall, significant improvements are necessary throughout the manuscript.

1) All experimental details regarding the preparation of 16S rRNA gene amplicons should be described, including the specific primer set, amplification conditions, and technical replicates. The manuscript lacks sufficient details for sequence analysis of the 16S rRNA gene. No considerations were made regarding data transformation or sequencing depth normalization. Potential confounding factors associated with RAI therapy were not assessed before performing between-group comparisons. In the co-occurrence networks, network properties or indices should be used to compare microbial networks between groups.
2) According to the reference Exact sequence variants should replace operational taxonomic units in marker-gene data analysis (2017) 11:2639-2643, amplicon sequence variant (ASV)-based species definitions are strongly recommended using DADA2, Deblur, or VSEARCH, rather than OTU-based definitions.
3) As this study is longitudinal, all data should be presented in a time-series manner, rather than as pairwise between-group comparisons.
4) The manuscript contains numerous inconsistencies between figures and their descriptions. For example:
- The figure captions for Figure 1 (e.g., panels 1b-1f) do not match the actual panel labels in the composite image.
- Heatmaps in Figures 2d, 2e, 3e, and 3f are presented without a labeled color scale.
- The main text describing Figures 4 and 5 is vague; specific panels are not cited.
- Lines 236-237 use raw taxonomic prefixes (e.g., p_Actinobacteria), which is inappropriate for a formal manuscript.
- Lines 255-257 state a "transient and significant change in richness on Day 3," which contradicts lines 223-224: "there were no significant differences in the α-diversity indices…."

5) Two arguments regarding the Clostridia are conflicting and unclear:
- NER analysis (lines 313-315) reports Clostridia enrichment in the NER group on Day 3, and lines 433-435 hypothesize a link to inflammation, suggesting antibiotics targeting Clostridia could enhance treatment efficacy.
- Main cohort analysis (lines 240-244) identifies genera such as Blautia and Anaerostipes-members of Clostridia-as beneficial bacteria that decreased on Day 3. The conclusion (lines 465-470) describes these as "SCFA-producing beneficial bacteria" and recommends probiotic supplementation.
6) Lines 366-369 report that "the antioxidant metabolite taurine decreased on Day 3 but recovered on Day 30," which underpins the recommendation for taurine supplementation in lines 469-470. However, Figures 3e and 3f do not clearly show that taurine levels on Day 30 returned to baseline levels from Day 0, and no statistical analysis is provided.

Reviewer #3 (Comments for the Author):

Dear Authors,

From line 81 to 138: "131 I" treatment wrongly appeared throughout instead of "RAI". Please correct this error.

Well done for the thorough job done in this study. I have a few comments below:
Line 348: "…Lachnospiraceae known for synthesis SCFAs by fermentation of dietary poly-saccharides"

Please correct the grammar error as follows: "...Lachnospiraceae known for the synthesis of SCFAs by fermentation of dietary polysaccharides"

Reviewer #4 (Comments for the Author):

This study presents a valuable longitudinal investigation into the dynamic alterations of the gut ecosystem during radioactive iodine (RAI) therapy. Nevertheless, several limitations warrant consideration to enhance the robustness of the findings and their interpretation.
1. The small sample size (n=25) restricts statistical power, thereby increasing the likelihood of Type II error. This limitation may hinder the detection of subtle yet biologically significant changes, potentially resulting in the omission of microbial or metabolomic markers with genuine predictive potential. As a single-center study, the patient cohort is likely to be homogeneous in terms of genetic background, dietary habits, and clinical management protocols. Therefore, the generalizability of the findings to broader populations, diverse ethnic groups, or healthcare systems with differing treatment strategies may be constrained.
2. The current analysis considers the three time points as independent observations, thereby reducing the study to a series of cross-sectional comparisons and precluding the assessment of longitudinal data trajectories. Implementing trajectory-based clustering methods on individual microbial and metabolite abundance profiles may facilitate the identification of distinct temporal response patterns.
3. The use of Linear Mixed Models (LMMs) is highly recommended, as these models more effectively account for individual variability and accommodate missing data in longitudinal studies. LMMs enable a direct assessment of the time-by-group interaction, thereby allowing evaluation of whether the rate of change-represented by the slope of the trajectory-differs significantly between the ER and NER groups.
4. Where feasible, a time-lagged correlation analysis is recommended. For example, evaluating the correlations between microbiota composition at Day 0 and metabolite profiles at Day 3 may provide preliminary insights into whether microbial changes precede and potentially drive subsequent metabolic alterations, or conversely, whether metabolic shifts influence microbial dynamics. This approach can offer more robust evidence for formulating mechanistic hypotheses regarding the role of the microbiota in modulating host metabolism and, ultimately, treatment efficacy.
5. The successful completion of the critical feature selection phase has facilitated an in-depth investigation into predictive modeling and patient heterogeneity, with differential taxa and metabolites systematically identified. To enhance the translational relevance of these findings, it is recommended to apply machine learning algorithms-specifically, LASSO regression for feature dimensionality reduction in combination with classification models such as Random Forest or Support Vector Machines-to integrate the selected biomarkers and rigorously evaluate their predictive performance for treatment response using metrics including AUC. This integrative strategy supports the transformation of distinct biological markers into a unified and clinically applicable composite scoring system.
6. Differences in treatment response may be attributable to pre-existing variations in the baseline gut microbial ecological composition. Enterotype analysis performed at baseline (Day 0) may facilitate patient stratification into distinct microbial community types, such as those characterized by dominance of Bacteroides or Prevotella.
7. The lack of mechanistic validation constitutes a significant limitation of the present study. All hypotheses regarding the potential roles of specific bacterial taxa or metabolites-such as the putative beneficial effects of Blautia or the radioprotective properties of taurine-are based solely on observational data and therefore remain speculative. In the absence of experimental confirmation, it is not possible to determine whether these microbial or metabolic features directly contribute to improved treatment outcomes or are merely correlative markers associated with other, as yet unidentified, causal factors.

Dear Editor,

We are very grateful for your kind letter. And we would like to express our thanks and appreciation for the reviewers' work. The comments and constructive suggestions are very helpful in further improving the quality of our manuscript.

Based on all comments and requests, we have made corresponding modifications to the original manuscript as listed below, and carefully proof-read the manuscript to minimize typographical and grammatical errors.

In order to facilitate the review of editors and reviewers, we have tracked changes in the revised manuscript and highlighted the added Supplemental Materials in red.

Replies to Reviewer #1:

1. The reviewer's comment:

   All experimental details regarding the preparation of 16S rRNA gene amplicons should be described, including the specific primer set, amplification conditions, and technical replicates. The manuscript lacks sufficient details for sequence analysis of the 16S rRNA gene.

   The authors' answer:

   Thank you for this important comment. We apologize for the incomplete presentation of the 16S rRNA sequencing analysis methodology. We will include the following methodological details in the Supplemental Materials (Page 1 Line 1-19).

   Supplemental sequencing method

   Total genome DNA from samples was extracted using CTAB/SDS method, with its concentration and purity monitored on 1% agarose gels. According to the concentration, DNA was diluted to 1ng/μl using sterile water. Amplification of the 16S rRNA gene V4 region was performed using barcoded primers 515F-806R in a 30 μL PCR reaction containing 15μL of Phusion® High-Fidelity PCR Master Mix (New England Biolabs), 0.2μM of forward and reverse primers, and about 10 ng template DNA.Thermal cycling consisted of initial denaturation at 98℃ for 1 min, followed by 30 cycles of denaturation at 98℃ for 10 s, annealing at 50℃ for 30 s,

and elongation at 72℃ for 30 s. Finally 72℃ for 5 min. Mix same volume of 1X loading buffer (contained SYB green) with PCR products and operate electrophoresis on 2% agarose gel for detection. Samples with bright main strip between 400-450bp were chosen for further experiments.

PCR products was mixed in equidensity ratios. Then, mixture PCR products was purified with GeneJET Gel Extraction Kit (Thermo Scientific). Sequencing libraries were generated using NEB Next® Ultra™ DNA Library Prep Kit for Illumina (NEB, USA) following manufacturer's recommendations and index codes were added. The library quality was assessed on the Qubit@ 2.0 Fluorometer (Thermo Scientific) and Agilent Bioanalyzer 2100 system. At last, the library was sequenced on an Illumina HiSeq platform and 250 bp paired-end reads were generated.

2. The reviewer's comment:

No considerations were made regarding data transformation or sequencing depth normalization.

The authors' answer:

Thank you for your important comment. In this study, we performed normalization on the raw data, and all downstream analyses were based on the normalized relative abundance data. This normalization effectively eliminated the potential influence of sequencing depth variation. As suggested, we have now added a clear statement in the main text Materials and Methods section (<u>Page 7 Line 147-148</u>) and have clarified in the relevant figure captions that the analyses were based on relative abundance (<u>Page 30 Line 682-683, 688-690</u>). We appreciate your comment, which has helped us improve the clarity and rigor of our methodological description.

Page 7 Line 147-148: As for bioinformatics analysis, the raw OTU counts were normalized to relative abundances to account for differences in sequencing depth.

Page 30 Line 682-683: Fig. 1 (e) Principal component analysis (PCA) based on relative abundance matrices at genus level. (f) Anosim analysis based on relative abundance matrices at genus level.

Page 30 Line 688-690: Fig. 2 (d) Heatmap of the relative abundances of top20 different flora at genus level among samples. (e) Heatmap of the relative abundances of top20 different flora at genus level among groups.

3. The reviewer's comment:

Potential confounding factors associated with RAI therapy were not assessed before performing between-group comparisons.

The authors' answer:

Thank you for highlighting this critical point. To minimize the potential interference of confounding factors between groups, we first conducted a balance test on the baseline clinical data of patients in both ER and NER groups, which included demographic characteristics and disease-related indicators, prior to conducting inter-group comparisons. The results confirmed the comparability of the two groups (Supplemental Materials Table S1), with no statistically significant differences observed in any of the measured indicators. This suggests that the gut microbiome and metabolite differences identified in subsequent analyses are more specifically associated with RAI treatment efficacy and are less likely to be influenced by other common clinical variables.

Table S1 Baseline clinical characteristics of included PTCRT patients.

**Baseline characteristics**	**PTCRT patients (n=24)**		***P* value**
	**ER (n=11)**	**NER (n=13)**	
Age, years, mean ± SD	44.64±9.04	38.77±8.91	0.124
Gender, *N* (%)			0.166
Male	1 (9.1%)	5 (38.5%)	
Female	10 (90.9%)	8 (61.5%)	
Risk level, *N* (%)			0.659
Medium	7 (63.6%)	10 (76.9%)	
High	4 (36.4%)	3 (23.1%)	

Clinical stage, *N* (%)			0.659
I	7 (63.6%)	10 (76.9%)	
II	4 (36.4%)	3 (23.1%)	
Maximum tumor diameter, cm, median (P25, P75)	1.30 (0.80, 2.25)	1.50 (0.90, 1.80)	0.794
Local invasion, *N* (%)			0.082
Present	5 (45.5%)	11 (84.6%)	
Absent	6 (54.5%)	2 (15.4%)	
Lymph node metastasis, *N* (%)			1.000
Present	10 (90.9%)	12 (92.3%)	
Absent	1 (9.1%)	1 (7.7%)	
Distant metastasis, *N* (%)			1.000
Present	0 (0.0%)	1 (7.7%)	
Absent	11 (100.0%)	12 (92.3%)	
sTg or TgAb level, *N* (%)			0.519
sTg < 1 ng/ml	3 (27.3%)	1 (7.7%)	
sTg 1-10 ng/ml	2 (18.2%)	3 (23.1%)	
sTg > 10 ng/ml or rising TgAb level	6 (54.5%)	9 (69.2%)	

PTCRT, papillary thyroid carcinoma after radical thyroidectomy; ER, excellent response; NER, non-excellent response; sTg, stimulated thyroglobulin; TgAb, thyroglobulin antibody.

4. The reviewer's comment:

   In the co-occurrence networks, network properties or indices should be used to compare microbial networks between groups.

   The authors' answer:

   Thank you for this kind suggestion. In our study, we have visualized the co-occurrence networks for each time point (Fig. 2g) and computed a full set of topological properties for comparison, with the complete data presented in Supplemental Materials Table S2.

Table S2 Quantitative co-occurrence network properties during RAI treatment.

	Nodes	Edges	Average degree	Graph density	Diameter	Average path length	Global transitivity
Day0	30	48	3.2	0.1103448	8	3.701149	0.3483871
Day3	32	35	2.1875	0.07056452	14	5.693548	0.15
Day30	24	74	6.166667	0.2681159	4	1.905797	0.4604651

In the revised manuscript, we will more prominently interpret the ecological implications of these property changes to clarify the dynamic shifts in microbial community disturbance and recovery (Page 13 Line 259-270) as following "In order to explore the symbiotic and competitive interactions within the microbial community during RAI treatment, we mapped the co-occurrence networks (Fig. 2g). Quantitative network properties were presented in Table S2. Compared to the baseline, the microbial network on Day3 became markedly sparser, evidenced by a visible decrease in both average degree and graph density, which signaled a sharp reduction of genera interactions. The structural fragmentation was accompanied by extended network diameter and average path length, indicating that information transfer within the community had become less efficient. A concurrent substantial drop in global transitivity further pointed to weakened stability. However, all these network properties robustly rebounded on Day30, with several reaching levels that surpassed the baseline. This indicates that microbial interactions had reconstituted into a stabilized network structure".

5. The reviewer's comment:

According to the reference Exact sequence variants should replace operational taxonomic units in marker-gene data analysis (2017) 11:2639-2643, amplicon sequence variant (ASV)-based species definitions are strongly recommended using DADA2, Deblur, or VSEARCH, rather than OTU-based definitions.

The authors' answer:

Thank you for this critical methodological suggestion. We have carefully reviewed

the recommended literature and fully acknowledge the superior resolution of ASV-based definitions. However, the primary focus of our study was explicitly on the genus-level taxonomy. At this broader taxonomic rank, the resolution offered by OTU clustering at 97% similarity is widely considered sufficient and remains a standard, accepted approach in the field, as evidenced by its continued use in recent publications (e.g., Decreased circulating IPA levels identify subjects with metabolic comorbidities: A multi-omics study. *Pharmacol Res.* 2024; 204:107207.; The relationship between the early-life gastrointestinal microbiome and childhood nocturnal cough. *J Allergy Clin Immunol.* 2025; 155(4): 1386-1391.).

Our entire experimental and analytical pipeline was designed and executed based on this OTU framework. Re-analyzing all data with an ASV-based definitions would constitute a fundamental redesign of the study's bioinformatic foundation, which is not feasible within the scope of this revision.

We sincerely feel sorry that we are unable to incorporate the suggested ASV-based approach in the current revision, but deeply appreciate the insightful and constructive suggestion. We fully acknowledge the methodological importance of ASVs and will certainly commit to prioritizing ASV-based definitions in future studies.

6. The reviewer's comment:

As this study is longitudinal, all data should be presented in a time-series manner, rather than as pairwise between-group comparisons.

The authors' answer:

Thank you for this kind suggestion. We would like to clarify that this study has already visualized the overall microbial community structure including both alpha and beta diversity (Fig. 1b-d, f), as well as PCA of microbiota and metabolites (Fig. 1e, Fig. 3a), microbial co-occurrence networks (Fig. 2g), and the relative abundances of the top20 genera and top30 metabolites (Fig. 2d-e, Fig. 3e-f) in a time-series manner. Together, these analyses reveal the overall dynamic trajectory of the microbial community and metabolites throughout the RAI treatment.

The presented pairwise comparisons (Day 0 vs. Day 3, Day 3 vs. Day 30) were conducted to focus on the two key transition phases induced by RAI therapy, thereby providing a more detailed interpretation of the macroscopic shifts observed in the time-series analysis and further clarifying the overall longitudinal fluctuation trend. We have also elaborated on this trend in the main text Discussion section (Page 17-20 Line 367-414).

7. The reviewer's comment:

The manuscript contains numerous inconsistencies between figures and their descriptions. For example:

- The figure captions for Figure 1 (e.g., panels 1b-1f) do not match the actual panel labels in the composite image.

The authors' answer:

We sincerely apologize for this oversight. We have thoroughly checked and corrected all figures to ensure complete consistency between the panels and their corresponding captions.

The reviewer's comment:

- Heatmaps in Figures 2d, 2e, 3e, and 3f are presented without a labeled color scale.

The authors' answer:

Thank you for your kind correction. We have confirmed that clear color-scale legends have been added to all revised heatmaps (Fig. 2d-e, 3e-f).

The reviewer's comment:

- The main text describing Figures 4 and 5 is vague; specific panels are not cited.

The authors' answer:

Thanks for your kind suggestion. We have revised the corresponding descriptions in the main text Results section (Page 15-16 Line 303-304, Line 317-319, Line 328-330) and confirmed that all figure citations in the text now clearly indicate the specific panels referenced.

Page 15 Line 303-304: The differential metabolites were mapped to the KEGG

database for the enrichment of functional modules (Fig. 4a-c) and metabolic pathways (Fig. 4d-f).

Page 15 Line 317-319: To explore the potential relationships between the alterations in gut microbiome and metabolome, a correlation matrix was generated using Spearman correlation between Day0 and Day3 (Fig. 5a), Day3 and Day30 (Fig. 5b), respectively.

Page 16 Line 328-330: The time-lagged correlation analysis (Fig. 5c) revealed a substantial number of significant microbiota-metabolite pairs across different time points, with a notable directional distribution.

The reviewer's comment:

- Lines 236-237 use raw taxonomic prefixes (e.g., p_Actinobacteria), which is inappropriate for a formal manuscript.

The authors' answer:

We apologize for the nonstandard terminology used. All informal prefixes such as "p_Actinobacteria" have been replaced with their correct taxonomic names (e.g., "Actinobacteria") throughout the manuscript.

The reviewer's comment:

- Lines 255-257 state a "transient and significant change in richness on Day 3," which contradicts lines 223-224: "there were no significant differences in the α-diversity indices...."

The authors' answer:

Thank you very much for this valuable comment. Alpha diversity indices reflect the overall richness and evenness of a community. These index results showing no statistical difference indicated that RAI treatment did not lead to a collapse of the overall microbial structure. In contrast, LEfSe analysis is aimed to identify specific taxa that are statistically and biologically discriminative between groups. The former perspective described macro-level structural stability, while the latter revealed fine-scale shifts in bacterial abundances (i.e., a decrease in certain taxa accompanied by an increase in others). They jointly demonstrated that the effect of

RAI was best characterized as microbial restructuring rather than broad-spectrum destruction. We have clarified this point in the main text (Page 11-12 Line 231-233, 243-244) to avoid potential misunderstanding.

Page 11 Line 231-233: Regardless of the genus or phylum level, there were no significant differences in the α-diversity indices of gut microbiota among three groups (Fig. 1b), indicating that RAI treatment did not lead to the collapse of the overall microbial community structure.

Page 12 Line 243-244: To further observe the specific differences in gut microbiota during RAI treatment, we performed LEfSe analysis.

8. The reviewer's comment:

Two arguments regarding the Clostridia are conflicting and unclear:

- NER analysis (lines 313-315) reports Clostridia enrichment in the NER group on Day 3, and lines 433-435 hypothesize a link to inflammation, suggesting antibiotics targeting Clostridia could enhance treatment efficacy.

- Main cohort analysis (lines 240-244) identifies genera such as Blautia and Anaerostipes-members of Clostridia-as beneficial bacteria that decreased on Day 3. The conclusion (lines 465-470) describes these as "SCFA-producing beneficial bacteria" and recommends probiotic supplementation.

The authors' answer:

Thank you for pointing out the key contradiction.

The ambiguity in the original statement stemmed from the generalized description of the heterogeneous definition "*Clostridia*", which may undergo complex functional restructuring within the class during RAI therapy. In the revised text, we have explicitly distinguished the components within the Discussion section. We specified that the fluctuating changes observed in the main cohort analysis referred specifically to the genus *Blautia* (Page 18 Line 380-382), while the clostridial subgroups enriched in the NER group may be associated with altered bile acid (BA) metabolism and a pro-inflammatory environment (Page 22 Line 473-477). Accordingly, we have restricted the precise identification and targeting strategies

to the specific BAs-producing Clostridial subgroups (Page 22-23 Line 478-480). Similarly, in the Conclusion section, the phrasing "some SCFAs-producing beneficial bacteria" (Page 24 Line 511-512) limited the scope of Clostridia members. Furthermore, the suggestion to supplement with probiotics was directed toward restoring functional taxa such as *Blautia* (Page 24 Line 514), thereby avoiding the potential conceptual conflation.

Page 18, Line 380-382: Furthermore, our study showed genus *Blautia*, which is belonging to family *Lachnospiraceae* known for the synthesis of SCFAs by fermentation of dietary polysaccharides, decreased on Day3. The specific amendments in the manuscript are as follows:

Page 22, Line 473-477: On Day3, *Clostridia* was found to enrich in NER patients. A published literature reported a *Clostridia*-rich microbiota as well as more bile acids (BAs) such as glycochenodeoxycholic acid and glycoursodeoxycholic acid in IBS patients, which reached an agreement with our observation (although these two BAs were not included in the top20 metabolites).

Page 22-23 Line 478-480: Given that *Clostridia*-derived BAs could promote intestinal inflammation, we assumed that precisely identifying and targeting these specific BAs-producing *Clostridial* subgroups could enhance the treatment efficacy of RAI.

Page 24 Line 510-515: This characterization could be summarized as "fluctuating alterations", where some SCFAs-producing beneficial bacteria and several antioxidant and anti-inflammatory substances reduced transiently, but opportunistic pathogens increased temporarily, suggesting that decent amounts of probiotics (e.g., *Blautia*) and anti-inflammatory and antioxidant metabolites might be beneficial for patients undergoing RAI treatment.

9. The reviewer's comment:

Lines 366-369 report that "the antioxidant metabolite taurine decreased on Day 3 but recovered on Day 30," which underpins the recommendation for taurine supplementation in lines 469-470. However, Figures 3e and 3f do not clearly show

that taurine levels on Day 30 returned to baseline levels from Day 0, and no statistical analysis is provided.

The authors' answer:

Thank you for the kind suggestion. We apologize for the incomplete presentation of information in the original figure. In response, we have supplemented a statistical plot showing paired sample comparisons of taurine levels across different time points in Fig. 3g, thereby providing rigorous validation of the conclusion that its level "returned to baseline".

The relative abundances of taurine were analyzed using a linear mixed-effects model, and the statistical outcomes were reported in the main text Results section (Page 14 Line 298-300). Taurine levels showed a significant decrease from Day0 to Day3 ($P < 0.01$). Although an increasing trend was observed from Day3 to Day30, the difference did not reach statistical significance, which may be due to the current limited sample size (Page 19 Line 401-402). These results indicate that taurine follows a trajectory of initial decline followed by recovery, with its level at Day30 stabilizing and returning close to the Day0 baseline ($P > 0.05$).

Furthermore, we have added analogous statistical plots based on linear mixed-effects models for other key bacterial genera and metabolites of interest (Fig. 2f, 3g). Corresponding descriptions have been included in the Results section of the main text (Page 12 Line 254-258, Page 14 Line 300-302).

Page 14 Line 298-300: Taurine showed a significant decline ($P = 0.024$) from Day0 to Day3, with its abundance recovering to baseline level by Day30 ($P > 0.05$), yet it did not exhibit a statistically significant overall fixed effect.

Page 19 Line 401-402: ...the lack of a significant fixed effect of taurine may be attributed to outliers in limited samples...

Page 12 Line 254-258: Fig. 2f demonstrated a significant fixed effect of time point on changing patterns of *Blautia* and *Streptococcus* ($P = 0.011$ and $P < 0.001$, respectively). For *Streptococcus* in particular, a significant trend of increasing and then decreasing was observed across both phases ($P = 0.003$ and $P < 0.001$, respectively).

Page 14 Line 300-302: Conversely, the changes in both FAHFA (2:0/20:2) and 4-HPA were characterized by a significant main effect of time (P = 0.004 and 0.049, respectively).

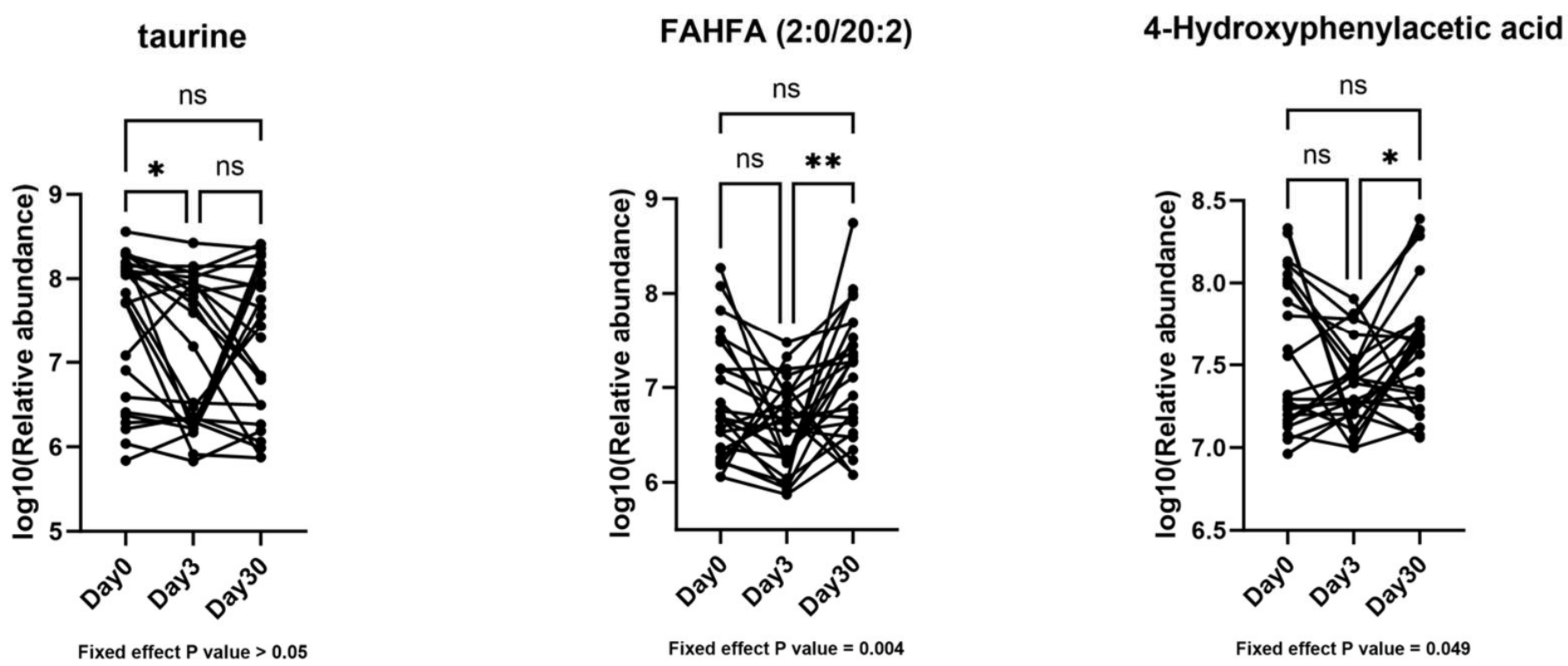

Fig. 3g Violin plots of the relative abundances of taurine, Fatty acid esters of hydroxy fatty acids (2:0/20:2), 4-Hydroxyphenylacetic acid across different time points.

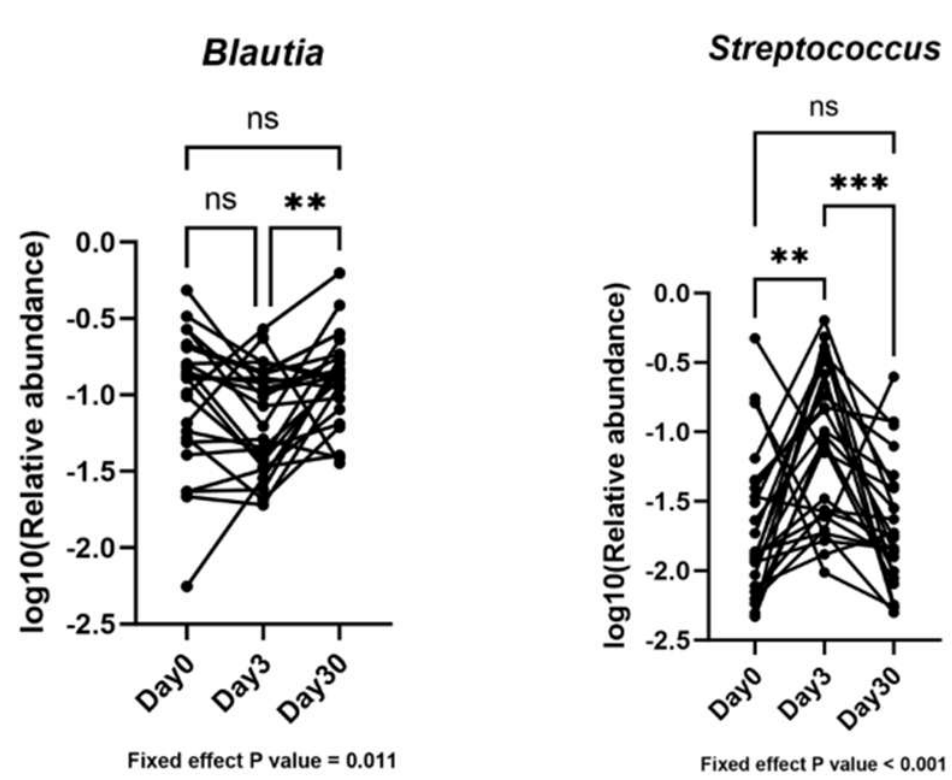

Fig. 2f Violin plots of the relative abundances of *Blautia* and *Streptococcus* across different time points.

Replies to Reviewer #3:

1. The reviewer's comment:

   Dear Authors,

   From line 81 to 138: "131 I" treatment wrongly appeared throughout instead of "RAI". Please correct this error.

   The authors' answer:

   Thank you for the kind correction. We sincerely apologize for this oversight. We have carefully reviewed the entire manuscript and have uniformly corrected all instances of the erroneous term "131 I treatment" to "RAI therapy" throughout the text.

2. The reviewer's comment:

   Well done for the thorough job done in this study. I have a few comments below:

   Line 348: "...Lachnospiraceae known for synthesis SCFAs by fermentation of dietary poly-saccharides". Please correct the grammar error as follows: "...Lachnospiraceae known for the synthesis of SCFAs by fermentation of dietary polysaccharides"

   The authors' answer:

   Thank you for the kind correction, and the grammatical error originally in Line 348 has been corrected as follows (Page 18 Line 381-382): "...*Lachnospiraceae* known for the synthesis of SCFAs by fermentation of dietary polysaccharides".

Replies to Reviewer #4:

1. The reviewer's comment:

   The small sample size (n=25) restricts statistical power, thereby increasing the likelihood of Type II error. This limitation may hinder the detection of subtle yet biologically significant changes, potentially resulting in the omission of microbial or metabolomic markers with genuine predictive potential. As a single-center study, the patient cohort is likely to be homogeneous in terms of genetic background, dietary habits, and clinical management protocols. Therefore, the generalizability of the findings to broader populations, diverse ethnic groups, or healthcare systems with differing treatment strategies may be constrained.

   The authors' answer:

   Thank you for the important comment. We fully acknowledge the validity of your point regarding sample size and study generalizability. As an exploratory longitudinal observation, this study faced practical challenges in patient recruitment and serial sample collection. We explicitly recognize that the current sample size (n=25) is relatively small, which limits statistical power and affects the generalizability of the findings. This is indeed a key limitation of our work, and we have clearly stated it in the main text Discussion section (Page 23 Lines 493-496) as "Certainly, there are some limitations in the current study. Firstly, although this study is the first of its kind to characterize the dynamic changes in gut microbiota and metabolites under internal radiation therapy, further validation of the findings is needed as it is a single-center, small-scale study.". Nonetheless, we believe our findings provide a preliminary foundation and a reference for future validation in large-sample and multi-center cohorts.

2. The reviewer's comment:

   The current analysis considers the three time points as independent observations, thereby reducing the study to a series of cross-sectional comparisons and precluding the assessment of longitudinal data trajectories. Implementing trajectory-based clustering methods on individual microbial and metabolite

abundance profiles may facilitate the identification of distinct temporal response patterns.

The authors' answer:

Thank you for your valuable suggestion regarding the assessment of longitudinal data trajectories. In response, we have supplemented the individual trajectory-based clustering analysis (Supplemental Materials Fig. S4, Table S3).

We first identified the top10 differential microbial genera between the ER and NER groups using LEfSe analysis, and the top10 differential metabolites via T-test. The longitudinal abundance trajectories of these key microbes and metabolites across the three time points were then modeled using k-means clustering. The optimal number of clusters (ranging from 2 to 5) for each variable was determined based on the silhouette coefficient. In this clustering analysis, the average silhouette coefficient was 0.535, indicating good overall clustering quality.

Subsequently, we assessed the association between these trajectory clusters and patient response to RAI therapy using the chi-square test or Fisher's exact test. Among the 20 variables analyzed, the longitudinal trajectory pattern of the metabolite N-Acetyldopamine showed a statistically significant correlation with treatment response ($P = 0.027$). The trajectory of N-Acetyldopamine was divided into five clusters, suggesting potential heterogeneity in its dynamic recovery process among patients, and this heterogeneity may be related to treatment outcome. In contrast, the trajectory clusters of other variables did not show significant associations with therapeutic response in this cohort.

Furthermore, the trajectory clustering analysis revealed that, compared to microbial genera, the longitudinal abundance patterns of metabolites exhibited a better ability to distinguish RAI treatment response. The analysis deepens our understanding of the longitudinal dynamics of the gut microbiome and metabolism, and N-Acetyldopamine will be prioritized as a potential temporal biomarker in our subsequent validation work.

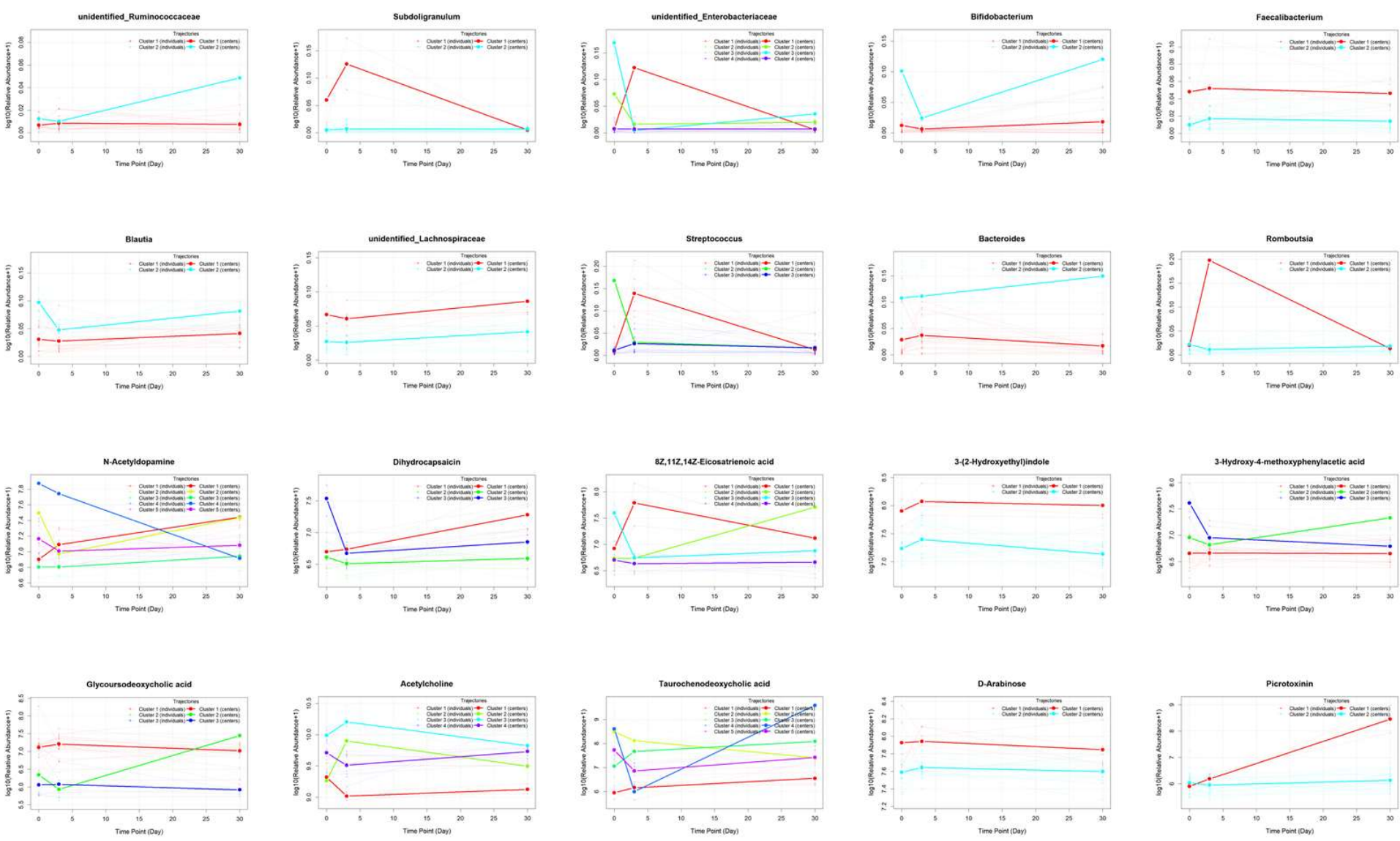

Fig. S4 Longitudinal trajectory clustering of top10 differential microbial genera and metabolic features.

Table S3 Results of longitudinal trajectory clustering for differential features and their association with RAI response.

Variable	Type	Best_Clusters	Avg_Silhouette	p_value
N-Acetyldopamine	metabolite	5	0.352	0.027
Dihydrocapsaicin	metabolite	3	0.471	0.071
8Z,11Z,14Z-Eicosatrienoic acid	metabolite	4	0.484	0.0783
3-(2-Hydroxyethyl)indole	metabolite	2	0.555	0.0815
3-Hydroxy-4-methoxyphenylacetic acid	metabolite	3	0.411	0.0893
Glycoursodeoxycholic acid	metabolite	3	0.481	0.1504
Acetylcholine	metabolite	4	0.433	0.177
Taurochenodeoxycholic acid	metabolite	5	0.369	0.1814
unidentified_Ruminococcaceae	microbiota	2	0.594	0.2229
D-Arabinose	metabolite	2	0.423	0.4245
Subdoligranulum	microbiota	2	0.849	0.4819
Picrotoxinin	metabolite	2	0.638	0.4819

unidentified_Enterobacteriaceae	microbiota	4	0.644	0.5715
Bifidobacterium	microbiota	2	0.700	0.5761
Faecalibacterium	microbiota	2	0.549	0.5963
Blautia	microbiota	2	0.408	0.751
unidentified_Lachnospiraceae	microbiota	2	0.467	0.813
Streptococcus	microbiota	3	0.534	0.8195
Bacteroides	microbiota	2	0.582	1
Romboutsia	microbiota	2	0.750	1

Thank you again for your important comment. We look forward to applying this methodological approach in future work to systematically investigate the asociations of a wider array of microbial taxa and metabolic features with RAI therapeutic outcomes.

3. The reviewer's comment:

The use of Linear Mixed Models (LMMs) is highly recommended, as these models more effectively account for individual variability and accommodate missing data in longitudinal studies. LMMs enable a direct assessment of the time-by-group interaction, thereby allowing evaluation of whether the rate of change-represented by the slope of the trajectory-differs significantly between the ER and NER groups.

The authors' answer:

Thank you for this valuable suggestion. Following your advice, we conducted an exploratory analysis using LMMs on the longitudinal data of 10 key differential microbial genera and 10 key differential metabolites between the ER and NER groups. This approach allows for a more rigorous assessment of whether the dynamic trends of gut microbiota and metabolites differ significantly between patients with different treatment responses.

In the models, the log-transformed value of each microbe or metabolite served as the dependent variable. Fixed effects included the time point, response group, and the crucial "time × response group" interaction term. A random intercept for each individual was included to account for within-subject correlation across

repeated measurements. Testing the statistical significance of this interaction term directly evaluated whether the slopes of change over time differ significantly between the two patient groups. The corresponding *P*-values were presented in Supplemental Materials Table S4.

The results showed that for all microbes and metabolites included, the "time × response group" interaction term did not reach statistical significance. The outcome indicated that although differences in certain microbes and metabolites were observed via LEfSe analysis or T-test, their longitudinal change trajectories could not currently serve as statistically significant markers for distinguishing RAI treatment response. Nonetheless, the application of LMMs to this longitudinal data provides a methodological reference for identifying dynamic biomarkers with differential trends between different response groups in larger future cohorts, and we will apply this approach in subsequent investigations.

Table S4 Assessment of the longitudinal trajectories of top10 differential microbial genera and metabolites between ER and NER groups via linear mixed-effects models.

Variable	type	P_value_raw	P_value_FDR
unidentified_Enterobacteriaceae	microbiota	0.077	0.821
Dihydrocapsaicin	metabolite	0.102	0.821
Bacteroides	microbiota	0.123	0.821
N-Acetyldopamine	metabolite	0.171	0.848
3-Hydroxy-4-methoxyphenylacetic acid	metabolite	0.374	0.848
D-Arabinose	metabolite	0.403	0.848
unidentified_Ruminococcaceae	microbiota	0.456	0.848
Subdoligranulum	microbiota	0.477	0.848
Acetylcholine	metabolite	0.522	0.848
Blautia	microbiota	0.526	0.848
8Z,11Z,14Z-Eicosatrienoic acid	metabolite	0.532	0.848

Streptococcus	microbiota	0.538	0.848
Romboutsia	microbiota	0.552	0.848
Picrotoxinin	metabolite	0.607	0.858
unidentified_Lachnospiraceae	microbiota	0.711	0.858
Faecalibacterium	microbiota	0.712	0.858
Bifidobacterium	microbiota	0.770	0.858
Taurochenodeoxycholic acid	metabolite	0.812	0.858
3-(2-Hydroxyethyl)indole	metabolite	0.815	0.858
Glycoursodeoxycholic acid	metabolite	0.950	0.950

4. The reviewer's comment:

Where feasible, a time-lagged correlation analysis is recommended. For example, evaluating the correlations between microbiota composition at Day 0 and metabolite profiles at Day 3 may provide preliminary insights into whether microbial changes precede and potentially drive subsequent metabolic alterations, or conversely, whether metabolic shifts influence microbial dynamics. This approach can offer more robust evidence for formulating mechanistic hypotheses regarding the role of the microbiota in modulating host metabolism and, ultimately, treatment efficacy.

The authors' answer:

Thank you for this valuable suggestion. Focusing on the top20 microbial genera and top30 metabolites that showed significant changes across time points in our prior analyses, we performed Spearman correlation analysis to investigate the cross-correlation of datasets across different time points. Since none of the *P*-values adjusted by the FDR method reached statistical significance, the exploratory analysis employed an uncorrected threshold of $P < 0.05$ to identify potential temporal correlations. The time-lagged correlation analysis revealed a substantial number of significant microbe-metabolite association pairs across different time intervals. Furthermore, the distribution of these associations exhibited a notable directional pattern. The microbe-metabolite association pairs with statistically

significant were displayed in Fig. 5c, and the detailed results (Page 16 Line 328-338) and the relevant discussion (Page 22 Line 460-469) were provided in the main text.

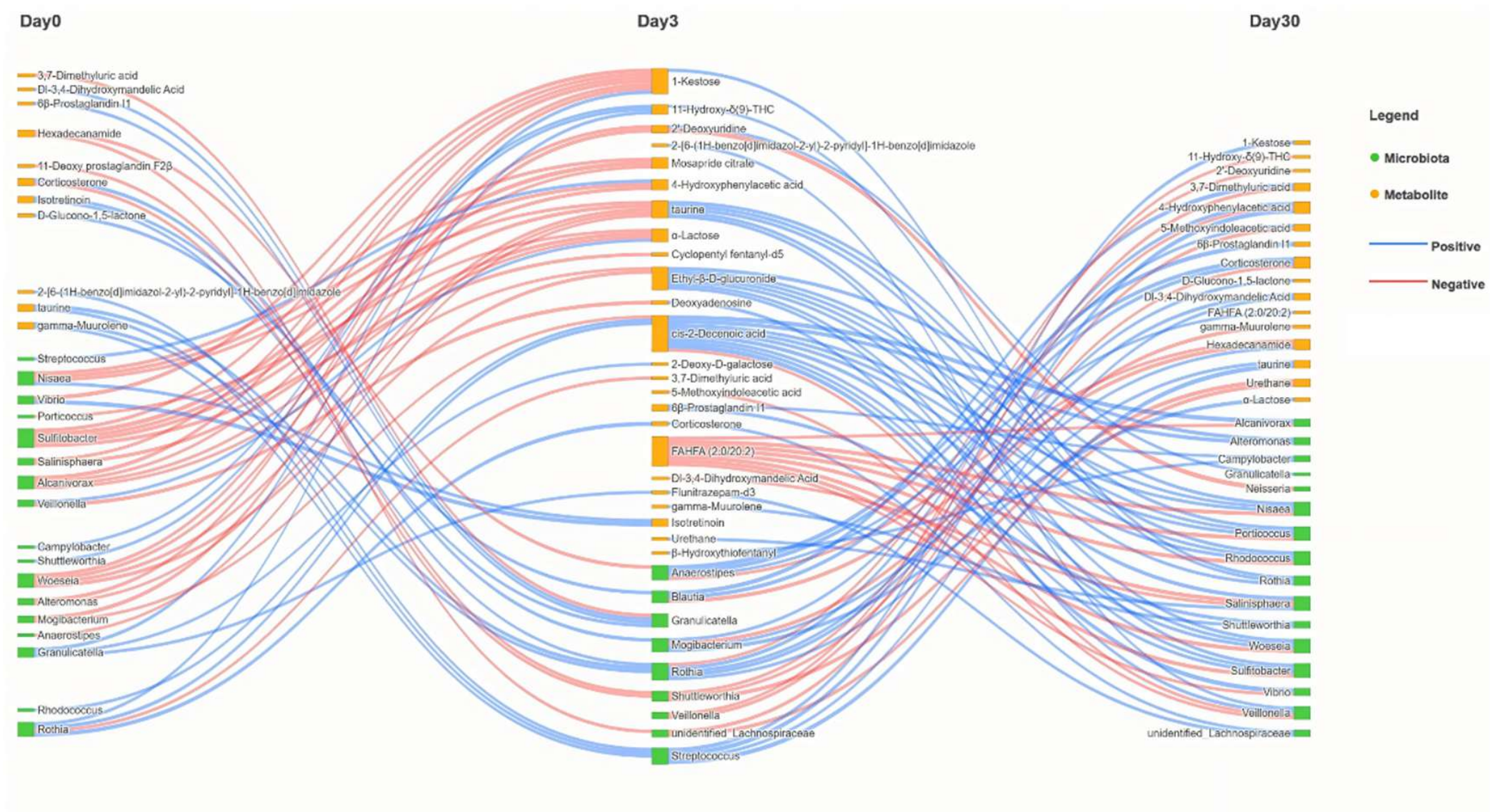

Fig. 5c Sankey diagram of time-lagged correlation between microbial genera across three time points. The correlation effect is indicated by a color gradient from blue (negative correlation) to red (positive correlation).

Page 16 Line 328-338: The time-lagged correlation analysis (Fig. 5c) revealed a substantial number of significant microbiota-metabolite pairs across different time points, with a notable directional distribution. Namely, 40 significant correlations were identified between Day0 microbiota and Day3 metabolite, whereas only 16 were found between Day0 metabolite and Day3 microbiota. Conversely, 27 significant pairs were observed between Day3 microbiota and Day30 metabolite, compared to 45 between Day3 metabolite and Day30 microbiota. These included a positive correlation between Streptococcus at Day0 and 4-hydroxyphenylacetic acid at Day3, as well as between Streptococcus at Day3 and 4-hydroxyphenylacetic acid at Day30. Furthermore, Blautia at Day3 was positively correlated with D-glucono-1,5-lactone at Day0, and with both 3,7-dimethyluric acid and corticosterone at Day30.

Page 22 Line 460-469: The time-lagged correlation analysis revealed the Day0

microbiota–Day3 metabolite associations predominated in the acute phase post-therapy. This indicates that gut microbial composition preceded or potentially shaped the subsequent metabolic profile during the initial perturbation. Interestingly, this directional pattern reversed in the later recovery period, with Day3 metabolite-Day30 microbiota pairs being more numerous. The shift may suggest that the earlier metabolic alterations thereafter played a decisive role in driving microbial community reconstruction. Intervention strategies targeting the gut microbiota in the acute phase and modulating the metabolic landscape during recovery period may promote the intestinal ecosystem rehabilitation.

In addition, we have refined the "Correlation analysis between gut microbiota and metabolites" section in the revised manuscript to better capture the dynamic co-variation between microbiota and metabolites. For each subject, we calculated the change values in bacterial genus abundance and metabolite concentration for the Day0–Day3 and Day3–Day30 intervals. Spearman correlations were performed between the paired change values. The modification shifted the analytical focus from static, cross-sectional associations to the coordinated dynamic change within each transition phase. The results (Page 15-16 Line 317-327) and discussion (Page 21-22 Line 437-459) based on this refined analysis were detailed in the main text.

Page 15-16 Line 317-327: To explore the potential relationships between the alterations in gut microbiome and metabolome … strong positive correlations were observed between Streptococcus and mosapride citrate.

Page 21-22 Line 437-459: Among the considerable relationships between the gut microbiome changes and metabolic product … supplementing anti-inflammatory and antioxidant metabolites might be a logical approach to promote the restoration of normal intestinal environment, but further research is needed.

5. The reviewer's comment:

The successful completion of the critical feature selection phase has facilitated an in-depth investigation into predictive modeling and patient heterogeneity, with

differential taxa and metabolites systematically identified. To enhance the translational relevance of these findings, it is recommended to apply machine learning algorithms-specifically, LASSO regression for feature dimensionality reduction in combination with classification models such as Random Forest or Support Vector Machines-to integrate the selected biomarkers and rigorously evaluate their predictive performance for treatment response using metrics including AUC. This integrative strategy supports the transformation of distinct biological markers into a unified and clinically applicable composite scoring system.

The authors' answer:

Thank you for your kind advice. The primary objective of this study was to systematically characterize the dynamic crosstalk between RAI-induced radiation and the gut microbiota, as well as the intrinsic fluctuation patterns of the microbiota following RAI therapy. Therefore, a predictive model was not included in the current manuscript. However, based on the differential microbial taxa and metabolic features identified in this study, we have already employed LASSO regression to screen for biomarkers, and have successfully developed a treatment response prediction model. A manuscript reporting the subsequent work is currently under review. We sincerely appreciate your valuable suggestion, which has provided a clear direction for our follow-up research.

6. The reviewer's comment:

Differences in treatment response may be attributable to pre-existing variations in the baseline gut microbial ecological composition. Enterotype analysis performed at baseline (Day 0) may facilitate patient stratification into distinct microbial community types, such as those characterized by dominance of Bacteroides or Prevotella.

The authors' answer:

Thank you for the valuable comment. Following your advice, we performed enterotype analysis on the 24 samples at Day0 to investigate whether the baseline

gut microbial community could be stratified into distinct enterotypes and to assess their potential association with RAI treatment response.

First, microbial features with an average relative abundance below 0.001 were filtered to reduce noise. Partitioning Around Medoids (PAM) clustering based on Bray-Curtis distance was then used to distinguish the samples, with the optimal cluster number (K) determined by the silhouette coefficient.

The analysis results showed that for K-values ranging from 2 to 5, the corresponding silhouette coefficients were 0.132, 0.162, 0.142, and 0.150, respectively. The maximum silhouette coefficient was only 0.162 (K = 3), which was lower than the commonly accepted threshold (silhouette coefficient > 0.25), indicating poor clustering performance (Supplemental Materials Fig. S5).

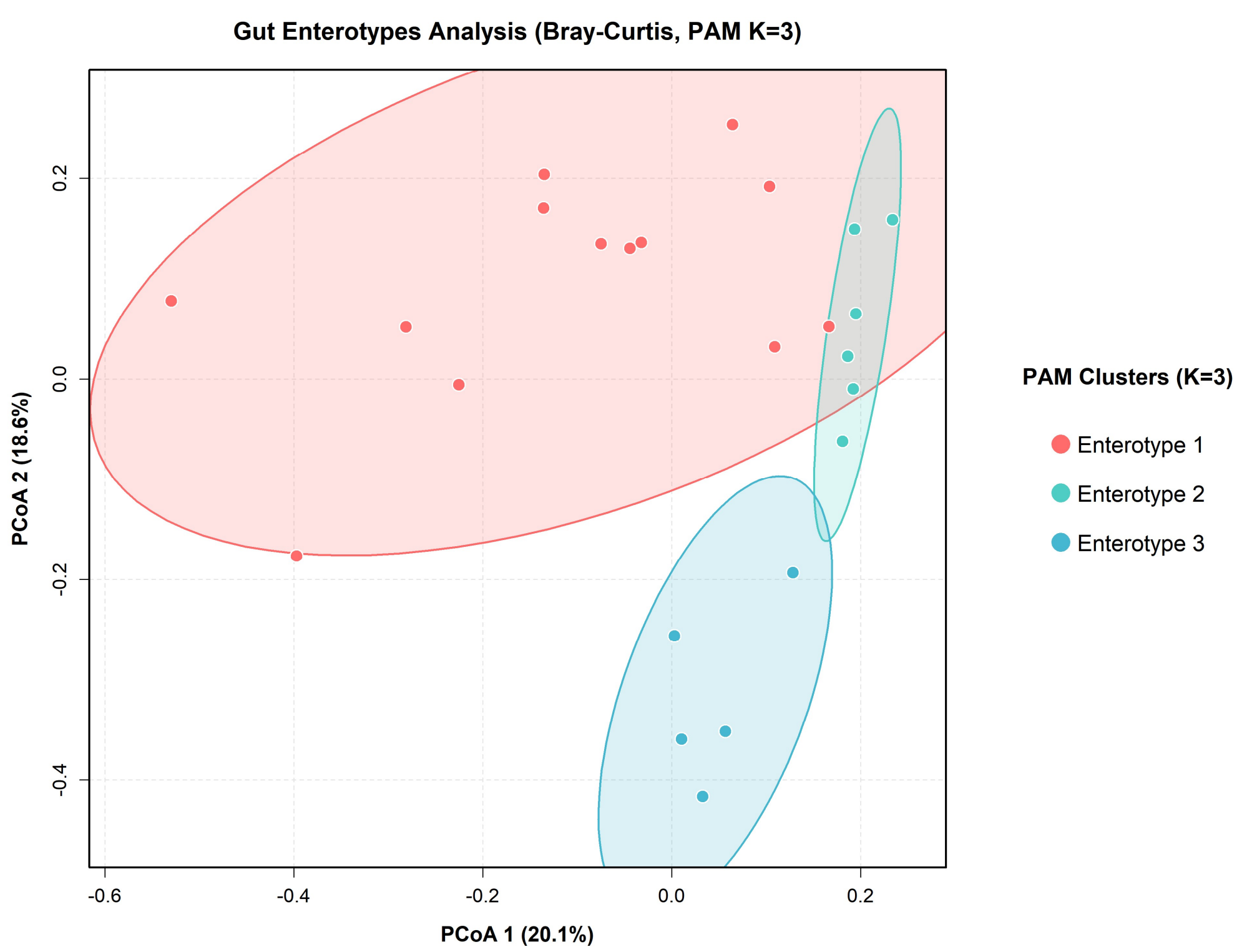

Fig. S5 Partitioning Around Medoids (PAM) clustering of baseline microbial composition into three enterotype groups showed poor clustering structure (silhouette coefficient = 0.162).

The clustering outcome suggested that the baseline microbial composition in our cohort did not form distinct enterotype clusters and could not be clearly classified into typical enterotypes such as *Bacteroides*-dominant or *Prevotella*-

dominant. This may reflect a highly homogeneous baseline gut microbial composition in our study cohort, possibly due to shared geographic origin and dietary patterns. Alternatively, it could indicate that enterotype stratification is not a key factor influencing treatment response.

Although the enterotype analysis did not result in clear clusters, we consider it an important methodological application that can be extended to future research. We are grateful for your suggestion, which prompted us to conduct further exploration.

7. The reviewer's comment:

The lack of mechanistic validation constitutes a significant limitation of the present study. All hypotheses regarding the potential roles of specific bacterial taxa or metabolites-such as the putative beneficial effects of Blautia or the radioprotective properties of taurine-are based solely on observational data and therefore remain speculative. In the absence of experimental confirmation, it is not possible to determine whether these microbial or metabolic features directly contribute to improved treatment outcomes or are merely correlative markers associated with other, as yet unidentified, causal factors.

The authors' answer:

Thank you for the kind opinion. We fully acknowledge that the "lack of mechanistic validation" represents a fundamental limitation of this observational study. As stated explicitly in the main text Discussion section (Page 23 Line 496-498) of "Secondly, this observational study lacks the elucidation of specific molecular mechanism or causal relationship, which requires subsequent intervention experiments.", all functional interpretations regarding microbes and metabolites were based on literature-supported correlative inferences and remained speculative at this stage, without establishing causality.

As this work remains an observational study and given the practical constraints on both experimentation and the revision timeline, it is not feasible to incorporate new interventional experiments at this stage. We entirely recognize this

limitation and plan to address it through experimental studies designed to functionally validate the underlying mechanisms in future work.

Re: Spectrum03237-25R1 (Dynamic effects of radioactive iodine therapy on gut microbiota and metabolites in patients with papillary thyroid cancer)

Dear Prof. Meng Liu:

Thank you for the privilege of reviewing your work. Below you will find my comments, instructions from the Spectrum editorial office, and the reviewer comments.

Please return the manuscript within 60 days; if you cannot complete the modification within this time period, please contact me. If you do not wish to modify the manuscript and prefer to submit it to another journal, notify me immediately so that the manuscript may be formally withdrawn from consideration by Spectrum.

Revision Guidelines
To submit your modified manuscript, log into the submission site at https://spectrum.msubmit.net/cgi-bin/main.plex. Go to Author Tasks and click the appropriate manuscript title to begin. The information you entered when you first submitted the paper will be displayed; update this as necessary. Note the following requirements:

• Upload point-by-point responses to the issues raised by the reviewers in a file named "Response to Reviewers," NOT in your cover letter.
• Upload a compare copy of the manuscript (without figures) as a "Marked-Up Manuscript" file.
• Upload a clean .DOC/.DOCX version of the revised manuscript and remove the previous version.
• Each figure must be uploaded as a separate, editable, high-resolution file (TIFF or EPS preferred), and any multipanel figures must be assembled into one file.
• Any supplemental material intended for posting by ASM should be uploaded with their legends separate from the main manuscript. You can combine all supplemental material into one file (preferred) or split it into a maximum of 10 files with all associated legends included.

For complete guidelines on revision requirements, see our Submission and Review Process webpage. Submission of a paper that does not conform to guidelines may delay acceptance of your manuscript.

Data availability: ASM policy requires that data be available to the public upon online posting of the article, so please verify all links to sequence records, if present, and make sure that each number retrieves the full record of the data. If a new accession number is not linked or a link is broken, provide Spectrum production staff with the correct URL for the record. If the accession numbers for new data are not publicly accessible before the expected online posting of the article, publication may be delayed; please contact production staff (Spectrum@asmusa.org) immediately with the expected release date.

Publication Fees: For information on publication fees and which article types are subject to charges, visit ourwebsite. If your manuscript is accepted for publication and any fees apply, you will be contacted separately about payment during the production process; please follow the instructions in that e-mail. Arrangements for payment must be made before your article is published.

ASM Membership: Corresponding authors may join or renew ASM membership to obtain discounts on publication fees. Need to upgrade your membership level? Please contact Customer Service at Service@asmusa.org.

The ASM Journals program strives for constant improvement in our submission and publication process. Please tell us how we can improve your experience by taking this quick Author Survey.

Thank you for submitting your paper to Spectrum.

Sincerely,
Wei-Hua Chen
Editor
Microbiology Spectrum

Reviewer #1 (Comments for the Author):

The revised manuscript is substantially improved compared to the original submission. However, the technical methods are still lacks critical details regarding sequencing procedures and data analysis. This is particularly important given that the study is observational in nature, meaning the validity of the results is heavily dependent on the appropriateness of the analytical approach.
Comment 1. The manuscript adequately describes the preparation of 16S rRNA gene amplicons; however, no information is provided regarding the preparation and handling of technical replicates for 16S rRNA gene amplicon sequencing.

Comment 2. The manuscript reports the use of total sum scaling (TSS) for sequencing depth normalization. While TSS can reduce sequencing depth-associated bias in beta-diversity analyses, it does not adequately address such bias in alpha-diversity analyses and between-group comparisons. Furthermore, prior to downstream analysis, potential false positives should be considered for exclusion from the dataset - including OTUs that are taxonomically unidentified, and OTUs with very low occurrence and/or abundance. None of these considerations are currently addressed in the manuscript.
Comment 3. A balance test was applied to identify potential confounding factors between groups. However, confounding should be further evaluated within both the microbiome and metabolome datasets, and appropriate adjustment for confounding factors should be applied in the microbiome and metabolome datasets.
Comment 4. Several methodological details remain missing or insufficiently described:
- No description is provided on how taxonomic lineages were identified.
- The bioinformatic tools used for sequence processing are not specified. The exact methods and software for sequence processing must be described in detail.
- Although the study focuses on genus-level taxonomy, many OTUs derived from amplicon sequencing data cannot be taxonomically classified to the genus level. It should be clearly described how such unclassified OTUs were handled in the analysis.

Reviewer #3 (Comments for the Author):

Dear Authors,

All review concerns raised have been sufficiently addressed.
Thank you!

Dear Editor,

We are very grateful for your kind letter. And we would like to express our thanks and appreciation for the reviewers' work. The comments and constructive suggestions are very helpful in further improving the quality of our manuscript.

Based on all comments and requests, we have made corresponding modifications to the original manuscript as listed below, and carefully proof-read the manuscript to minimize typographical and grammatical errors. In order to facilitate the review of editors and reviewers, we have tracked changes in the revised manuscript and highlighted the added Supplemental Materials in red.

Replies to Reviewer #1:

1. The reviewer's comment:

   The manuscript adequately describes the preparation of 16S rRNA gene amplicons; however, no information is provided regarding the preparation and handling of technical replicates for 16S rRNA gene amplicon sequencing.

   The authors' answer:

   Thank you for raising this important question. We have consulted the sequencing company (Novogene Company, China) and would like to provide the following clarification.

   In terms of the setup of technical replicates, we confirmed with Novogene that, according to their standard operating procedures, each sample undergoes only one round of DNA extraction, one library preparation, and one sequencing run. We sincerely appreciate your critical observation, and in future studies we will prioritize the inclusion of technical replicates by explicitly requesting that a proportion of samples be subjected to repeated DNA extraction or library preparation, thereby further enhancing the reliability and reproducibility of our data.

   Although technical replicates were not included in this study, we emphasize that our experimental design relied on biological replicates (n = 25 per group), which capture natural inter-individual variation.

In addition, multiple quality control measures were implemented during the experimental and analytical processes to ensure data reliability. According to Novogene's experimental records, negative controls were included in each PCR batch using nuclease-free water from the commercial kit to monitor potential contamination during amplification. DNA extraction was performed using a standardized CTAB method across all samples, and PCR amplification was carried out with the same batch of Phusion® High-Fidelity PCR Master Mix under consistent thermal cycling conditions with fixed cycle numbers. Library construction was performed using the TruSeq® DNA PCR-Free Sample Preparation Kit to maintain uniformity. Sequencing was completed on a single flow cell of the Illumina NovaSeq 6000 platform to avoid batch effects that might arise from sequencing across multiple runs. In the bioinformatics stage, rigorous quality control was applied to the raw sequencing data, including sequence assembly using FLASH (V1.2.7), quality filtering with QIIME using a Q20 threshold, chimera removal with VSEARCH (V2.9.0) against the SILVA database, and rarefaction to normalize sequencing depth across samples. These standardized procedures and stringent quality control measures have been routinely adopted by Novogene in numerous published studies (1, 2), confirming that their sequencing pipeline conforms to industry standards. Through these steps, technical variability was minimized to the greatest extent possible.

2. The reviewer's comment:

The manuscript reports the use of total sum scaling (TSS) for sequencing depth normalization. While TSS can reduce sequencing depth-associated bias in beta-diversity analyses, it does not adequately address such bias in alpha-diversity analyses and between-group comparisons. Furthermore, prior to downstream analysis, potential false positives should be considered for exclusion from the dataset - including OTUs that are taxonomically unidentified, and OTUs with very low occurrence and/or abundance. None of these considerations are currently addressed in the manuscript.

The authors' answer:

Thank you for your valuable comments on our manuscript. We have carefully addressed each of your concerns and revised the manuscript accordingly. Our point-by-point responses are as follows.

- Regarding the issue of sequencing depth normalization

We fully agree with your point that relative abundances obtained by total sum scaling (TSS) have certain limitations in alpha diversity analyses and between-group comparisons. Therefore, we have processed the raw OTU count data using rarefaction and recalculated all alpha diversity indices based on the rarefied absolute abundances. The analyzed results are consistent with our original conclusions, but the methodological rigor has been significantly improved. We have updated **Fig. 1b** and made the corresponding modifications in the Materials and Methods section (Page 8 Line 160-165).

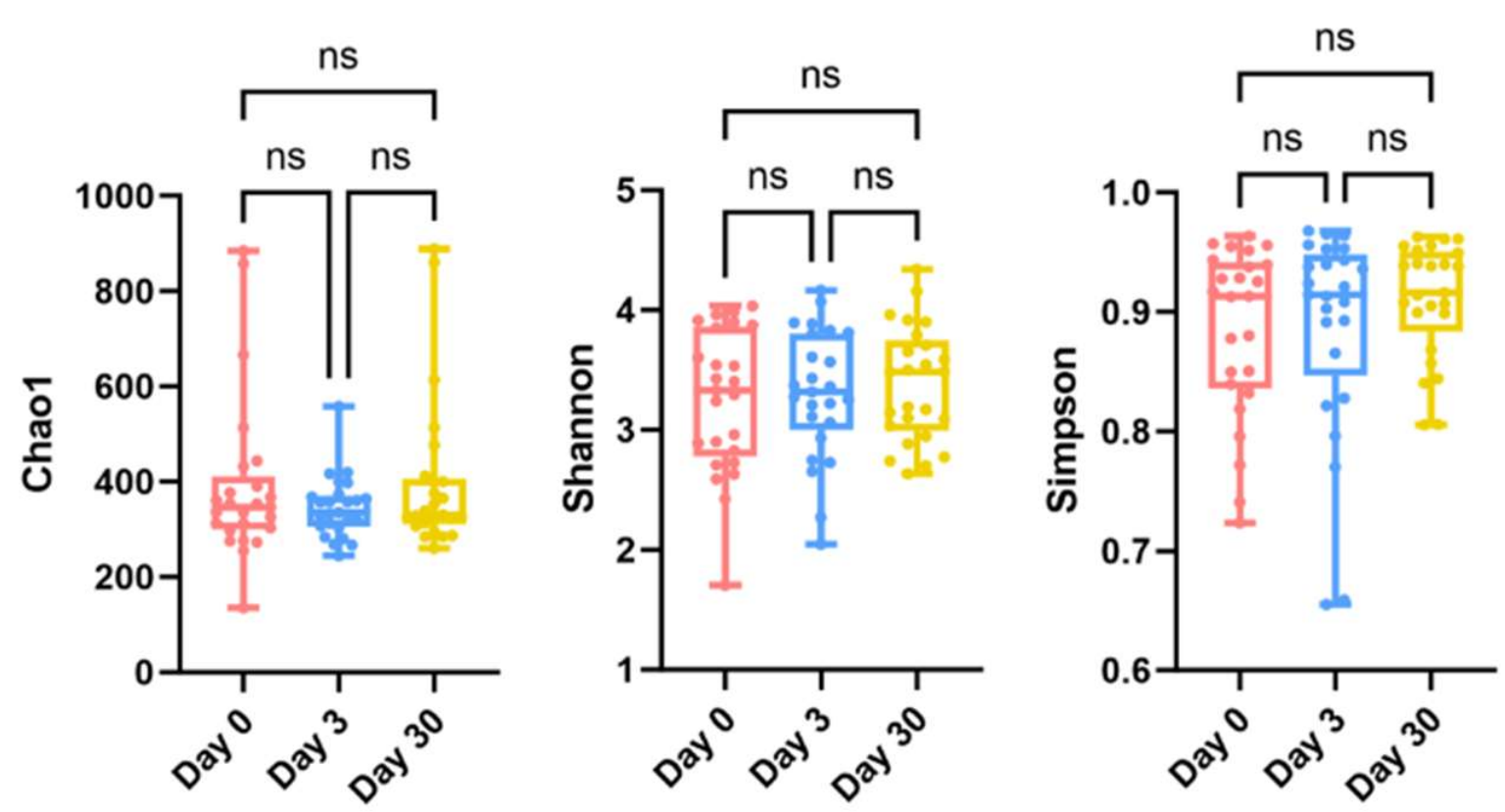

Fig. 1b The α diversity analysis based on the rarefied absolute abundances.

Page 8 Line 160-165: The raw OTU counts were then rarefied to an even sequencing depth to account for differences in sequencing depth across samples. Alpha (α) diversity indices were calculated based on the rarefied OTU table using R phyloseq (V1.34.0) and microbiome (V1.12.0) packages. Beta (β) diversity was assessed using Bray-Curtis distances calculated from relative abundance data obtained by total sum scaling (TSS) of the raw OTU counts.

At the genus level, we used the Wilcoxon-Mann-Whitney test to compare the relative abundances of OTUs between each pair of the three groups (Day0, Day3, and Day30), and genera with $P < 0.05$ were considered significantly different. Published microbiome studies have also used the Wilcoxon-Mann-Whitney test on relative abundance data for between-group comparisons (3, 4). In the heatmap presenting the top20 differential genera, if a genus showed $P < 0.05$ in multiple comparisons, we ranked it according to its smallest *P*-value. We have added the relevant description in the Materials and Methods section (Page 8-9 Line 171-175). In terms of identifying differential microbial taxa, we used LEfSe analysis to identify statistically significant biomarker taxa among groups. This method is by default based on relative abundance data and employs Kruskal-Wallis test combined with linear discriminant analysis to screen for differential taxa (5).

Page 8-9 Line 171-175： At the genus level, the Wilcoxon Mann Whitney test was used to compare the relative abundances of OTUs between each pair of the three groups (Day0, Day3, and Day30). Genera with $P < 0.05$ in at least one comparison were considered significantly different. For genera significant in multiple comparisons, the smallest *P*-value was used for ranking. The top20 genera with the smallest *P*-values were presented in the heatmap.

- Regarding the exclusion of potential false-positive OTUs

Before OTU clustering, we performed stringent quality control on all raw sequences and removed singletons, effectively reducing false-positive OTUs introduced by sequencing or PCR errors. After clustering, we applied multiple filtering steps to the OTUs. Specifically, we excluded OTUs that appeared in a single sample across the entire dataset, retaining only those with broader prevalence to ensure sufficient biological representation in the analysis. For taxonomic annotation, OTUs that could not be assigned to the next lower taxonomic level but could be annotated to a higher level, for example unclassified at the genus level but assignable to a family, were labeled as

"unidentified" and retained at the corresponding higher level. OTUs that could not be assigned to any taxonomic rank were directly discarded. These steps ensured the reliability of the data used for downstream analyses. We have added the above methodological details in the Materials and Methods section (Page 7 Line 147-149, Page 8 Line 154-155, Page 8 Line 157-160).

Page 7 Line 147-149: As for bioinformatics analysis, the entire workflow was conducted within the QIIME framework. Raw paired-end reads generated on an Illumina NovaSeq platform were first quality controlled, and singletons were discarded.

Page 8 Line 154-155: After clustering, OTUs that were detected only once across all samples were discarded.

Page 8 Line 157-160: OTUs unclassifiable at a given taxonomic level but assignable to a higher level were labeled as "unidentified" and kept at that higher level, while OTUs with no taxonomic assignment were excluded.

3. The reviewer's comment:

A balance test was applied to identify potential confounding factors between groups. However, confounding should be further evaluated within both the microbiome and metabolome datasets, and appropriate adjustment for confounding factors should be applied in the microbiome and metabolome datasets.

The authors' answer:

Thank you for raising this important point, which has helped us carefully evaluate potential confounding factors in our analyses.

To control for confounding, we applied linear mixed models (LMMs) with adjustments for age, sex, time point, and sequencing batch. In the microbiome analysis, a total of 75 samples were sequenced in five separate batches. Therefore, we included age, sex, and batch as fixed effects, and time point (Day0/Day3/Day30) as the primary independent variable of interest in the LMM. This allowed us to assess the effect of time on microbial abundance while

adjusting for potential confounders. Although a batch effect was observed for some taxa, principal coordinate analysis (PCoA) based on Bray-Curtis distances did not reveal clear clustering of samples by batch (**Fig. S1**), indicating that the batch effect was overall limited. Importantly, the time main effects of the two genera of our primary interest, *Blautia* and *Streptococcus*, remained statistically significant in the LMM after full adjustment (**Table S1**), confirming that our core findings are robust and not confounded by batch effects.

For the metabolome analysis, all samples were processed in a single batch. Thus, no batch effect was present. We included age, sex, and time point as fixed effects in the LMMs, and the time main effects of the key metabolites taurine and 4-hydroxyphenylacetic acid were still statistically significant (**Table S2**). The above methods and results for confounding control have been added to the revised manuscript (Page 9 Line 186-190, Page 11 Line 216-219, Page 13-14 Line 278-283, Page 15-16 Line 321-327). Accordingly, **Fig. 2f and 3g** have been updated.

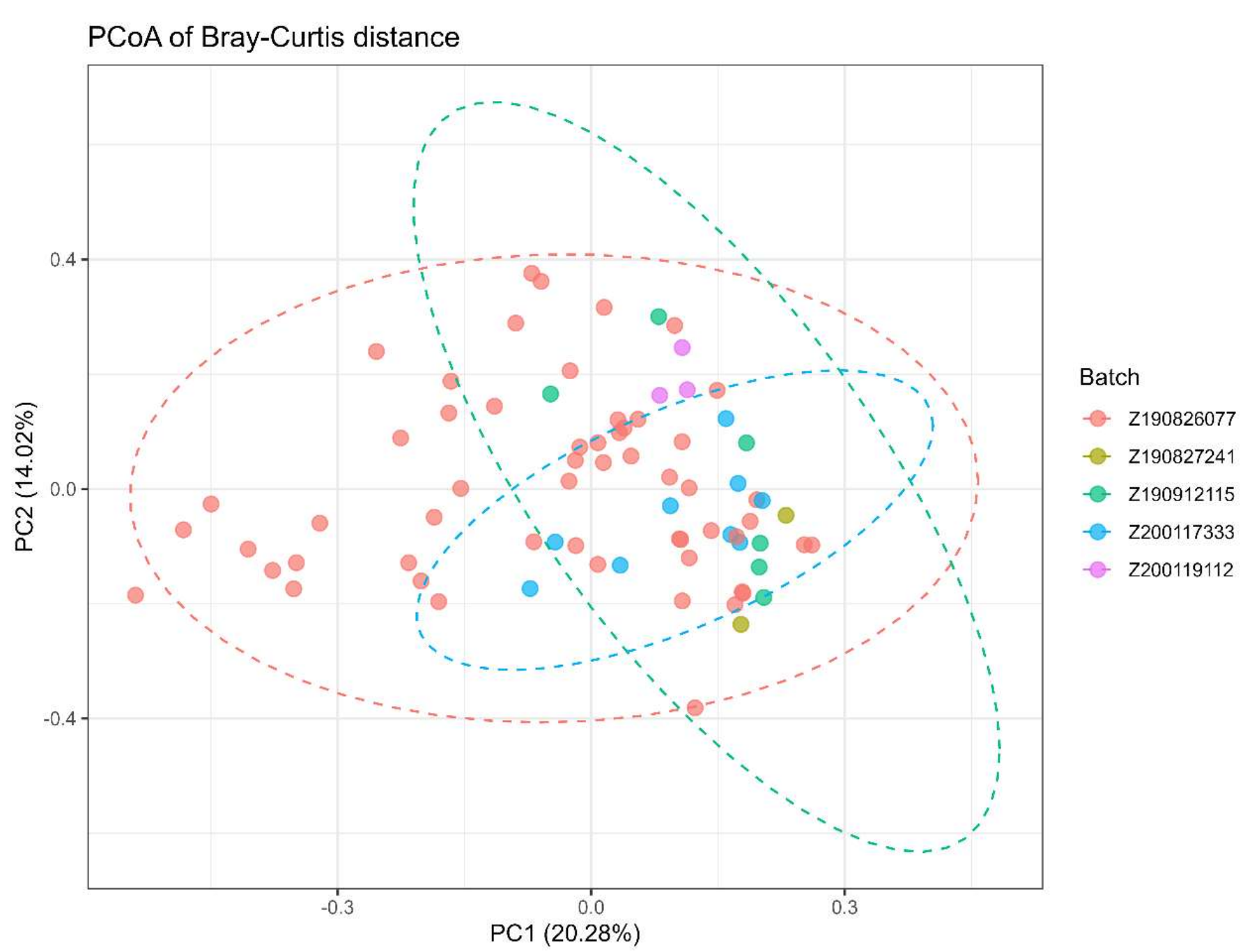

Fig. S1 Principal coordinate analysis (PCoA) of microbial communities based on Bray-Curtis distances, with samples colored by sequencing batch.

Table S1 Fixed effects of time, age, gender, and sequencing batch on each microbial feature estimated by linear mixed-effects models (LMMs).

Feature	Time_p	Age_p	Gender_p	Batch_p
unidentified_Lachnospiraceae	0.017	0.652	0.626	0.216
Anaerostipes	0.068	0.262	0.898	0.116
Blautia	0.025	0.611	0.493	0.220
Campylobacter	0.067	0.434	0.076	0.121
Shuttleworthia	0.539	0.144	0.274	0.002
Sulfitobacter	0.170	0.910	0.574	0.397
Woeseia	0.087	0.956	0.680	0.430
Alteromonas	0.113	0.492	0.964	0.686
Vibrio	0.174	0.596	0.822	0.784
Alcanivorax	0.147	0.763	0.715	0.614
Nisaea	0.318	0.726	0.575	0.364
Porticoccus	0.177	0.978	0.243	0.294
Rhodococcus	0.174	0.864	0.562	0.280
Salinisphaera	0.278	0.897	0.582	0.482
Granulicatella	0.183	0.744	0.075	0.585
Streptococcus	<0.001	0.903	0.825	0.924
Veillonella	0.038	0.464	0.908	0.012
Rothia	0.005	0.911	0.998	0.169
Mogibacterium	0.057	0.508	0.886	0.086
Neisseria	0.102	0.885	0.413	0.861

Table S2 Fixed effects of time, age and gender on each metabolite feature estimated by linear mixed-effects models (LMMs).

Feature	Time_p	Age_p	Gender_p
11-Hydroxy-δ(9)-THC	<0.001	0.035	0.836
Deoxyadenosine	0.077	0.909	0.972
2'-Deoxyuridine	0.089	0.499	0.018
2-Deoxy-D-galactose	0.088	0.583	0.387
taurine	0.108	0.156	0.320
3-(4-hydroxy-3-methoxyphenyl)propanoic acid	0.016	0.174	0.490
cis-2-Decenoic acid	0.001	0.285	0.474
Urethane	0.197	0.789	0.915
Flunitrazepam-d3	0.005	0.146	0.450
α-Lactose	0.040	0.966	0.898
Isotretinoin	<0.001	0.064	0.619
β-Hydroxythiofentanyl	0.002	0.386	0.340
Ethyl-β-D-glucuronide	0.119	0.228	0.183
D-Glucono-1,5-lactone	0.023	0.721	0.969
1-Kestose	0.313	0.281	0.447
3,7-Dimethyluric acid	0.066	0.537	0.838

11-Deoxy prostaglandin F2 β	0.051	0.015	0.617
2-[6-(1H-benzo[d]imidazol-2-yl)-2-pyridyl]-1H-benzo[d]imidazole	0.047	0.127	0.665
4-Hydroxyphenylacetic acid	0.043	0.194	0.194
Dl-3,4-Dihydroxymandelic Acid	0.013	0.026	0.175
Mosapride citrate	<0.001	0.551	0.891
Cyclopentyl fentanyl-d5	<0.001	0.360	0.705
Hexadecanamide	<0.001	0.878	0.686
5-Methoxyindoleacetic acid	<0.001	0.869	0.237
Corticosterone	0.022	0.067	0.140
Equol	0.189	0.773	0.916
6 β -Prostaglandin I1	0.052	0.747	0.952
FAHFA (2:0/20:2)	0.003	0.240	0.844
gamma-Muurolene	0.003	0.355	0.978
Hexadecanedioic acid	0.181	0.379	0.900

Page 9 Line 186-190: Linear mixed effects models (LMMs) were employed to account for potential confounding factors. The fixed effects included age, sex, time point, and sequencing batch. Pairwise comparisons were adjusted using Tukey's method for multiple testing. Principal coordinate analysis (PCoA) based on Bray Curtis distances was performed to visualize potential batch effects.

Page 11 Line 216-219: All samples were processed in a single batch, thus no batch effect was present. LMMs with age, sex, and time point as fixed effects were applied to longitudinally analyze the changes in key metabolites over time. Pairwise comparisons were performed with Tukey's correction.

Page 13-14 Line 278-283: Although a moderate batch effect was observed for some taxa (Fig. S1, Table S2), Fig. 2f demonstrated a significant fixed effect of time point on changing patterns of *Blautia* and *Streptococcus* ($P = 0.025$ and $P < 0.001$, respectively) after adjusting for potential confounding factors. For *Streptococcus* in particular, a significant trend of increasing and then decreasing was observed across both phases, with $P < 0.001$ for each pairwise comparison.

Page 15-16 Line 321-327: The biphasic trends in certain anti-inflammatory metabolites (FAHFA (2:0/20:2), 4-HPA and taurine) were presented in Fig. 3g. The changes in both FAHFA (2:0/20:2) and 4-HPA were characterized by a significant time fixed effect (P = 0.003 and 0.043, respectively), with a notable decrease from Day0 to Day3 followed by a further rally from Day3 to Day30.

Taurine displayed an analogous trajectory, yet its time main effect did not reach statistical significance in the LMM model.

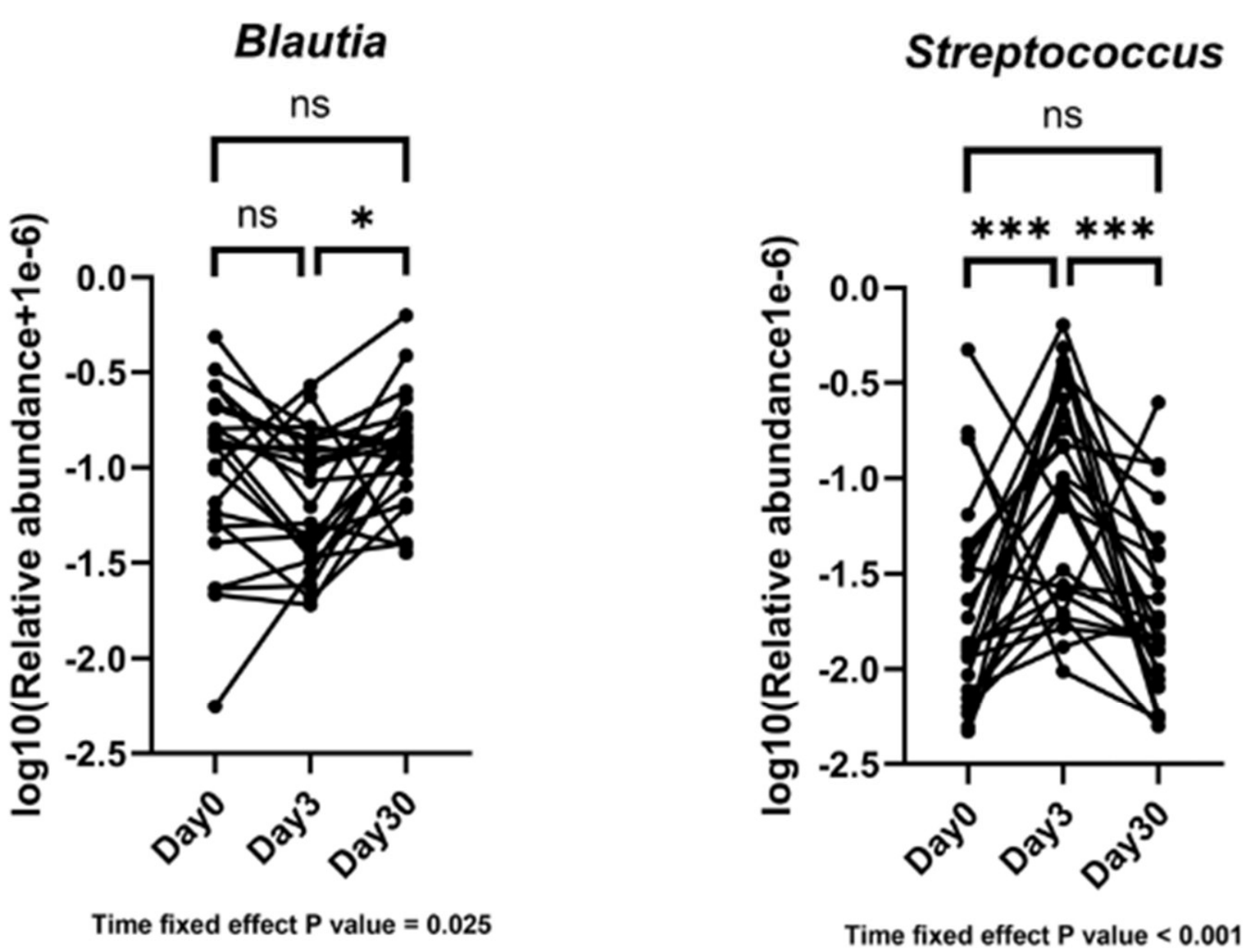

Fig. 2f Violin plots of the relative abundances of *Blautia* and *Streptococcus* across different time points. *P*-values were calculated using linear mixed effects models (LMMs) with adjustment for age, sex, time point, and sequencing batch.

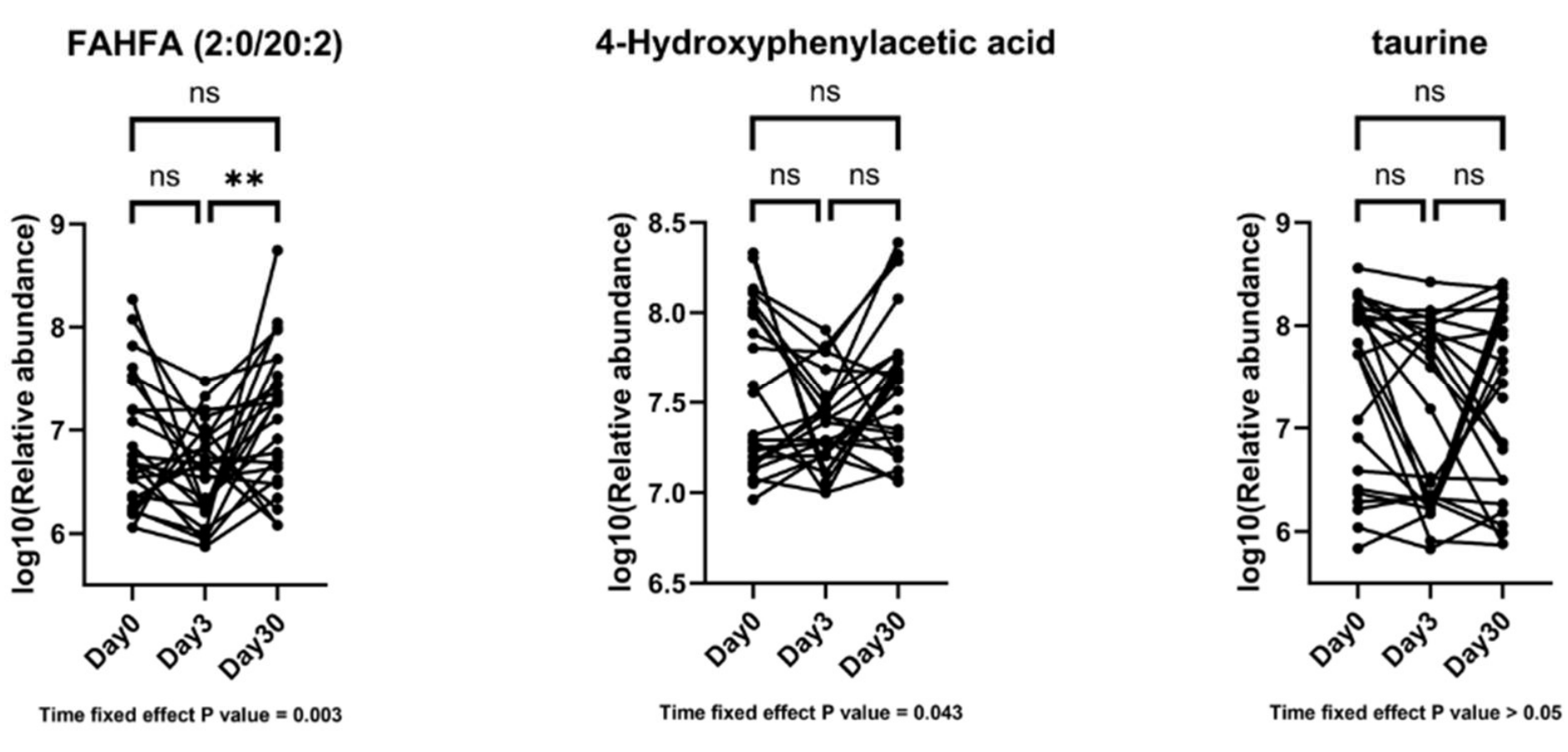

Fig. 3g Violin plots of the relative abundances of Fatty acid esters of hydroxy fatty acids (2:0/20:2), 4-Hydroxyphenylacetic acid and taurine across different time points. *P*-values were calculated using linear mixed effects models (LMMs) with adjustment for age, sex and time point.

4. The reviewer's comment:

Several methodological details remain missing or insufficiently described:

- No description is provided on how taxonomic lineages were identified.

- The bioinformatic tools used for sequence processing are not specified. The exact methods and software for sequence processing must be described in detail.

- Although the study focuses on genus-level taxonomy, many OTUs derived from amplicon sequencing data cannot be taxonomically classified to the genus level. It should be clearly described how such unclassified OTUs were handled in the analysis.

The authors' answer:

Thank you for your important comments. We have addressed each of your concerns based on our analytical workflow as follows.

- Regarding the identification of taxonomic lineages

We applied a sequence alignment strategy based on the hypervariable regions of the 16S rDNA. The representative sequence of each OTU was aligned against the SSU rRNA reference sequences in the SILVA132 database using Mothur. A bootstrap confidence threshold of 0.8 was applied (if an OTU failed to reach this confidence at a given taxonomic level, the algorithm recursively evaluated higher levels until the threshold was met). When the similarity between an unknown sequence and a reference sequence reached or exceeded this threshold, the sequence was assigned to the corresponding taxonomic level from phylum to genus.

- Regarding the bioinformatics tools and software used for sequence processing

Raw sequencing data were generated on an Illumina HiSeq platform, producing 250 bp paired-end reads. Quality filtering was performed using fastp with the parameters "-A -g -q 19 -u 15". Paired-end reads were then assembled into raw tags using FLASH (V1.2.7). Chimeric sequences were identified and removed using VSEARCH (V2.9.0) against the SILVA database as reference.

OTU clustering was carried out with Uparse (V7.0.1001) at 97% sequence identity, and the most abundant sequence within each OTU was selected as the representative sequence. Species annotation was performed using Mothur against the SILVA132 database with a confidence threshold of 0.8. The entire analysis workflow was conducted within the QIIME framework.

- Regarding the handling of OTUs that could not be classified to the genus level

Some OTUs could not be precisely assigned to a genus because their similarity to reference sequences in the SILVA database fell below the genus level threshold or because the database lacked appropriate reference sequences at the genus level. As mentioned in the response to the second question, we adopted a stratified strategy. If an OTU could be annotated to the family level but not to any genus, its taxonomic information was labeled as "unidentified" (e.g., *unidentified_Lachnospiraceae*) and the OTU was retained. If an OTU could not be annotated to any level from family upward, it was directly removed from downstream analyses.

All the above methodological details and the software used for sequence processing have been clearly described in the Materials and Methods section of the revised manuscript (Page 7-8 Line 147-160).

Page 7-8 Line 147-160: As for bioinformatics analysis, the entire workflow was conducted within the QIIME framework. Raw paired-end reads generated on an Illumina NovaSeq platform were first quality controlled, and singletons were discarded. The reads were then assembled and further quality filtered using fastp (-A -g -q 19 -u 15) and FLASH (V1.2.7) to obtain Clean Tags. Chimeric sequences were identified and removed with VSEARCH (V2.9.0) against the SILVA database, yielding Effective Tags. All Effective Tags were clustered into Operational Taxonomic Units (OTUs) at 97% sequence identity using Uparse (V7.0.1001). After clustering, OTUs that were detected only once across all samples were discarded. For taxonomic annotation, the retained OTU

representative sequences were performed with Mothur against the SSU rRNA reference sequences in the SILVA132 database using a confidence threshold of 0.8 OTUs unclassifiable at a given taxonomic level but assignable to a higher level were labeled as “unidentified” and kept at that higher level, while OTUs with no taxonomic assignment were excluded.

Thank you and all the reviewers for the kind advice again. We acknowledge the reviewers’ comments and suggestions very much, which are valuable in improving the quality of our manuscript.

Sincerely yours,

Yuhui Cao, Meng Liu, Huaiqiu Zhu

Reference

1. Chen J, Hu S, Ji D, Gao Z, Wang H, Yang Y, Chen Y, Gu J. 2020. Hemolysin BL from novel *bacillus toyonensis* BV-17 induces antitumor activity both in vitro and in vivo. Gut Microbes 12:1782158.

2. Li C, Li X, Guo R, Ni W, Liu K, Liu Z, Dai J, Xu Y, Abduriyim S, Wu Z, Zeng Y, Lei B, Zhang Y, Wang Y, Zeng W, Zhang Q, Chen C, Qiao J, Liu C, Hu S. 2023. Expanded catalogue of metagenome-assembled genomes reveals resistome characteristics and athletic performance-associated microbes in horse. Microbiome 11:7.

3. Yu L, Deng Y-H, Huang Y-H, Ke H-J, Guo Y, Wu J-L. 2021. Comparison of gut microbiota between infants with atopic dermatitis and healthy controls in guangzhou, China. JAA Volume 14:493–500.

4. Xu Q, Gill S, Xu L, Gonzalez E, Pichichero ME. 2019. Comparative analysis of microbiome in nasopharynx and middle ear in young children with acute otitis media. Front Genet 10:1176.

5. Erawijantari PP, Mizutani S, Shiroma H, Shiba S, Nakajima T, Sakamoto T, Saito Y, Fukuda S, Yachida S, Yamada T. 2020. Influence of gastrectomy for gastric cancer treatment on faecal microbiome and metabolome profiles. Gut 69:1404–1415.

Re: Spectrum03237-25R2 (Dynamic effects of radioactive iodine therapy on gut microbiota and metabolites in patients with papillary thyroid cancer)

Dear Prof. Meng Liu:

Your manuscript has been accepted, and I am forwarding it to the ASM production staff for publication. Your paper will first be checked to make sure all elements meet the technical requirements. ASM staff will contact you if anything needs to be revised before copyediting and production can begin. Otherwise, you will be notified when your proofs are ready to be viewed.

Data Availability: ASM policy requires that data be available to the public upon online posting of the article, so please verify all links to sequence records, if present, and make sure that each number retrieves the full record of the data. If a new accession number is not linked or a link is broken, provide production staff with the correct URL for the record. If the accession numbers for new data are not publicly accessible before the expected online posting of the article, publication may be delayed; please contact ASM production staff immediately with the expected release date.

Publication Fees: For information on publication fees and which article types have charges, please visit our website. We have partnered with Copyright Clearance Center (CCC) to collect author charges. If fees apply to your paper, you will receive a message from no-reply@copyright.com with further instructions. For questions related to paying charges through RightsLink, please contact CCC at ASM_Support@copyright.com or toll free at +1-877-622-5543. CCC makes every attempt to respond to all emails within 24 hours.

ASM Membership: Corresponding authors may join or renew ASM membership to obtain discounts on publication fees. Need to upgrade your membership level? Please contact Customer Service at Service@asmusa.org.

Embargo Policy: A press release may be issued as soon as the manuscript is posted on the Spectrum Latest Articles webpage. The corresponding author will receive an email with the subject line "ASM Journals Author Services Notification" when the article is available online.

The ASM Journals program strives for constant improvement in our submission and publication process. Please tell us how we can improve your experience by taking this quick Author Survey.

Thank you for submitting your paper to Spectrum.

Sincerely,
Wei-Hua Chen
Editor
Microbiology Spectrum
