# Supplementary material for: Dynamic effects of radioactive iodine therapy on gut microbiota and metabolites in patients with papillary thyroid cancer
Source: Microbiol Spectr. 2026 Jul 10;14(8):e03237-25. doi: 10.1128/spectrum.03237-25 (PMC13436337; doi:10.1128/spectrum.03237-25)
Supplement: Supplementary file 2 [file spectrum.03237-25-s0001.docx]

**Supplemental sequencing method**

Total genome DNA from samples was extracted using CTAB/SDS method, with its concentration and purity monitored on 1% agarose gels. According to the concentration, DNA was diluted to 1ng/μL using sterile water. Amplification of the 16S rRNA gene V4 region was performed using barcoded primers 515F-806R in a 30 μL PCR reaction containing 15μL of Phusion® High-Fidelity PCR Master Mix (New England Biolabs), 0.2μM of forward and reverse primers, and about 10 ng template DNA. Thermal cycling consisted of initial denaturation at 98℃ for 1 min, followed by 30 cycles of denaturation at 98℃ for 10 s, annealing at 50℃ for 30 s, and elongation at 72℃ for 30s. Finally, 72℃ for 5 min. Mix same volume of 1X loading buffer (contained SYB green) with PCR products and operate electrophoresis on 2% agarose gel for detection. Samples with bright main strip between 400-450bp were chosen for further experiments.

PCR products were mixed in equidensity ratios. Then, mixture PCR products were purified with GeneJET Gel Extraction Kit (Thermo Scientific). Sequencing libraries were generated using NEB Next® Ultra™ DNA Library Prep Kit for Illumina (NEB, USA) following manufacturer’s recommendations and index codes were added. The library quality was assessed on the Qubit@ 2.0 Fluorometer (Thermo Scientific) and Agilent Bioanalyzer 2100 system. At last, the library was sequenced on an Illumina HiSeq platform and 250 bp paired-end reads were generated.

**Table S1** Fixed effects of time, age, gender, and sequencing batch on each microbial feature estimated by linear mixed‑effects models (LMMs).

| Feature | Time_p | Age_p | Gender_p | Batch_p |
| --- | --- | --- | --- | --- |
| unidentified_Lachnospiraceae | 0.017 | 0.652 | 0.626 | 0.216 |
| Anaerostipes | 0.068 | 0.262 | 0.898 | 0.116 |
| Blautia | 0.025 | 0.611 | 0.493 | 0.220 |
| Campylobacter | 0.067 | 0.434 | 0.076 | 0.121 |
| Shuttleworthia | 0.539 | 0.144 | 0.274 | 0.002 |
| Sulfitobacter | 0.170 | 0.910 | 0.574 | 0.397 |
| Woeseia | 0.087 | 0.956 | 0.680 | 0.430 |
| Alteromonas | 0.113 | 0.492 | 0.964 | 0.686 |
| Vibrio | 0.174 | 0.596 | 0.822 | 0.784 |
| Alcanivorax | 0.147 | 0.763 | 0.715 | 0.614 |
| Nisaea | 0.318 | 0.726 | 0.575 | 0.364 |
| Porticoccus | 0.177 | 0.978 | 0.243 | 0.294 |
| Rhodococcus | 0.174 | 0.864 | 0.562 | 0.280 |
| Salinisphaera | 0.278 | 0.897 | 0.582 | 0.482 |
| Granulicatella | 0.183 | 0.744 | 0.075 | 0.585 |
| Streptococcus | <0.001 | 0.903 | 0.825 | 0.924 |
| Veillonella | 0.038 | 0.464 | 0.908 | 0.012 |
| Rothia | 0.005 | 0.911 | 0.998 | 0.169 |
| Mogibacterium | 0.057 | 0.508 | 0.886 | 0.086 |
| Neisseria | 0.102 | 0.885 | 0.413 | 0.861 |

**Table S2** Quantitative co-occurrence network properties during RAI treatment.

|  | Nodes | Edges | Average degree | Graph density | Diameter | Average path length | Global transitivity |
| --- | --- | --- | --- | --- | --- | --- | --- |
| Day0 | 30 | 48 | 3.2 | 0.1103448 | 8 | 3.701149 | 0.3483871 |
| Day3 | 32 | 35 | 2.1875 | 0.07056452 | 14 | 5.693548 | 0.15 |
| Day30 | 24 | 74 | 6.166667 | 0.2681159 | 4 | 1.905797 | 0.4604651 |

**Table S3** Fixed effects of time, age and gender on each metabolite feature estimated by linear mixed‑effects models (LMMs).

| Feature | Time_p | Age_p | Gender_p |
| --- | --- | --- | --- |
| 11-Hydroxy-δ(9)-THC | <0.001 | 0.035 | 0.836 |
| Deoxyadenosine | 0.077 | 0.909 | 0.972 |
| 2'-Deoxyuridine | 0.089 | 0.499 | 0.018 |
| 2-Deoxy-D-galactose | 0.088 | 0.583 | 0.387 |
| taurine | 0.108 | 0.156 | 0.320 |
| 3-(4-hydroxy-3-methoxyphenyl)propanoic acid | 0.016 | 0.174 | 0.490 |
| cis-2-Decenoic acid | 0.001 | 0.285 | 0.474 |
| Urethane | 0.197 | 0.789 | 0.915 |
| Flunitrazepam-d3 | 0.005 | 0.146 | 0.450 |
| α-Lactose | 0.040 | 0.966 | 0.898 |
| Isotretinoin | <0.001 | 0.064 | 0.619 |
| β-Hydroxythiofentanyl | 0.002 | 0.386 | 0.340 |
| Ethyl-β-D-glucuronide | 0.119 | 0.228 | 0.183 |
| D-Glucono-1,5-lactone | 0.023 | 0.721 | 0.969 |
| 1-Kestose | 0.313 | 0.281 | 0.447 |
| 3,7-Dimethyluric acid | 0.066 | 0.537 | 0.838 |
| 11-Deoxy prostaglandin F2β | 0.051 | 0.015 | 0.617 |
| 2-[6-(1H-benzo[d]imidazol-2-yl)-2-pyridyl]-1H-benzo[d]imidazole | 0.047 | 0.127 | 0.665 |
| 4-Hydroxyphenylacetic acid | 0.043 | 0.194 | 0.194 |
| Dl-3,4-Dihydroxymandelic Acid | 0.013 | 0.026 | 0.175 |
| Mosapride citrate | <0.001 | 0.551 | 0.891 |
| Cyclopentyl fentanyl-d5 | <0.001 | 0.360 | 0.705 |
| Hexadecanamide | <0.001 | 0.878 | 0.686 |
| 5-Methoxyindoleacetic acid | <0.001 | 0.869 | 0.237 |
| Corticosterone | 0.022 | 0.067 | 0.140 |
| Equol | 0.189 | 0.773 | 0.916 |
| 6β-Prostaglandin I1 | 0.052 | 0.747 | 0.952 |
| FAHFA (2:0/20:2) | 0.003 | 0.240 | 0.844 |
| gamma-Muurolene | 0.003 | 0.355 | 0.978 |
| Hexadecanedioic acid | 0.181 | 0.379 | 0.900 |

**Fig. S1** Principal coordinate analysis (PCoA) of microbial communities based on Bray‑Curtis distances, with samples colored by sequencing batch.

**Fig. S2** The differential metabolites in each enriched metabolic pathway of significance between Day0 and Day3. (a) The differential metabolites in Arginine and proline metabolism. (b) The differential metabolites in Folate biosynthesis. (c) The differential metabolites in Fluid shear stress and atherosclerosis. (d) The differential metabolites in Taste transduction. The annotated metabolites were indicated by solid green circles, the up-regulated differential metabolites on Day3 compared to Day0 were indicated by circles with red rims, and the down-regulated differential metabolites on Day3 compared to Day0 were indicated by circles with blue rims.

**Fig. S3** The differential metabolites in each enriched metabolic pathway of significance between Day3 and Day30. (a) The differential metabolites in Biosynthesis of unsaturated fatty acids. (b) The differential metabolites in Folate biosynthesis. The annotated metabolites were indicated by solid green circles, the up-regulated differential metabolites on Day30 compared to Day3 were indicated by circles with red rims, and the down-regulated differential metabolites on Day30 compared to Day3 were indicated by circles with blue rims.

**Fig. S4** The differential metabolites in enriched metabolic pathway of significance (Sulfur relay system) between Day0 and Day30. The annotated metabolites were indicated by solid green circles, the up-regulated differential metabolites on Day30 compared to Day0 were indicated by circles with red rims.
